# Companion animal owner “types” identified using a large-scale international assessment of the human-animal bond

**DOI:** 10.3389/fvets.2026.1748135

**Published:** 2026-05-12

**Authors:** Danny Maupin, Christos Dadousis, Anthony D. Whetton, Nophar Geifman

**Affiliations:** 1 School of Health Sciences, Faculty of Health and Medical Sciences, University of Surrey, Guildford, United Kingdom; 2 Veterinary Health Innovation Engine, School of Veterinary Medicine and School of Biosciences, Faculty of Health and Medical Sciences, University of Surrey, Guildford, United Kingdom; 3 School of Biosciences and Medicine, Faculty of Health and Medical Sciences, University of Surrey, Guildford, United Kingdom

**Keywords:** animal companion, cat, dog, human animal relationship, one health

## Abstract

**Introduction:**

Research into the human-animal bond has identified a complex relationship between pet ownership and health with both positive and negative effects. However, research into this bond has been limited by relatively small, heterogenous datasets. This study aims to define strata of cat and dog owners based on a large, multi-national dataset.

**Methods:**

Data was provided by the International Survey of Pet Owners and Veterinarians, encompassing information on 19,187 dog and cat owners across 10 countries. Strata of cat and dog owners were identified using a model-based clustering approach, with optimal number of clusters identified based on Bayesian Information Criterion (BIC) noting each cluster should contain at least 10% of overall population. Clusters were compared and contrasted across proportion of question responses (*χ*^2^ test) and mean response (Mann–Whitney U, Kruskal-Wallis tests).

**Results:**

Two disparate clusters were identified in dog owners, while three were identified in cat owners. Clusters were associated with different pet ownership characteristics, with subgroups of pet owners (Cluster 2 in dog owners, Clusters 2 and 3 in cat owners) that reported a strong bond with their pet, higher likelihood to spend more money and utilize veterinary products, and a stronger impact on their health. Gender was found to differ between clusters, with female owners tending to report a stronger bond wither their pet.

**Discussion:**

Our data-driven approach identified heterogeneity not only between owners of different pet types, but also within owners of the same pet type. Broadly, there appears to be a subgroup of owners with stronger emotional connections with their pet (report the pet is like a child, a stronger bond) compared to owners with a more pragmatic view of their pet. However, results do suggest a high baseline level of care that all owners provide for their pets.

## 1 Introduction

The enduring relationship between humans and domestic animals is well documented, extending at least as far back as 7,000 BP in Hemudu culture (1) and 3,000 BP in ancient Mesopotamia (2). Today, there are an estimated 10 million cats (23% of households with one or more cat) and 11.5 million dogs (30% of households with one or more dog) kept as pets in the UK (3), with similar rates of ownership found across Europe, Australia, China and Japan (4). These relationships show potential to offer substantial benefits to human health, ranging from serious mental health conditions including intellectual disability (5) and autism (6), to healthcare and rehabilitation (7, 8) and general well-being including both physical (9) and mental health (10). However, research has also highlighted negative components to pet ownership including practical and financial burdens to owning a pet as well as the psychological impact of a companion animal’s death (10), an impact that is observed in adults and children (11). These findings suggest a complex relationship between pet ownership and health-, particularly mental health, outcomes.

The concept of human-animal interactions, termed the human-animal bond, is a fairly recent area of research starting in the 1980s (12). Previous literature has investigated the relationship between the human-animal bond and human mental health, with one study reporting a curvilinear relationship, suggesting that extremely weak or strong bonds may be associated with a reduced capacity to build resilience, defined as a dynamic process that enables an individual to effectively negotiate, adapt to and/or manage sources of stress (13). A systematic review undertaken by Ellis et al. (10) further demonstrated this complexity, identifying 14 studies that have found no significant difference in depression between pet and non-pet owners. Further, it was found that studies measuring attachment bond revealed a positive or a non-significant relationship with depression, while those with high levels of secure attachment (i.e., lower attachment anxiety and attachment avoidance) were associated with lower levels of depression (10). Complicating interpretation of these findings is the high level of study heterogeneity, and risk of bias in studies, as reported on a state of assessment piece on human-animal interaction research (14), and discussed in systematic reviews (10, 15).

In addition to the wide variability in studies to date, any research on the impacts of the human-animal bond is further complicated by the likely heterogeneity within the studied human populations. Current research looking into contributing factors to the strength of the human-animal bond have focused on areas such as ethnic diversity (16, 17) or have assessed multiple factors including age, income, and sex (18, 19). Nevertheless, these studies have not addressed wider heterogeneity and are limited in part by convenience samples (e.g., attendees of an animal welfare symposium (18)) or have relatively moderate sample sizes (with a maximum of 587 participants seen (16)) across one or two countries.

The aim of this study was to take a data-driven approach to identifying and defining strata of cat and dog owners. Our objective was to provide robust insight into the human-animal bond and its impact across a range of constructs including pet-owners well-being, approach to veterinary care, and budget limitations for companion animal care. To do this we have employed a multi-national dataset across 10 countries and approximately 19,000 participants. We have identified substrata of companion animal guardians using a machine learning approach to the questionnaire data.

## 2 Methods

### 2.1 Data collection

Data were obtained from the International Survey of Pet Owners and Veterinarians (ISPOV) (20), commissioned by the Human Animal Bond Research Institute (HABRI) (21) in partnership with animal health company, Zoetis. This data was collected as part of a non-academic market survey. Participants provided informed consent, noting that their identity would remain confidential and that the information would be used for research purposes only. The data was anoymised prior to being shared with the research team. A total of 19,187 dog and cat owners and 1,512 small animal veterinarians from across 10 countries, including Australia, Brazil, China, France, Germany, Japan, Mexico, Spain, United Kingdom, and United States, were surveyed. Data collection started in 2021, with a second collection in 2022 that added extra responses from Mexico, and a third in 2023 that added extra responses from Australia. Participants were the primary caretakers of their companion animal, or pet, and the sample was balanced in respect of gender, age, and region. In the case of multiple pet households, participants were asked to focus on only one of their pets. Only pet-owners of cats or dogs were included in the survey.

Data was cleaned, removing missing or unreported demographic data (e.g., “prefer not to say” gender category) due to small sample sizes, as well as those with a HABSCORE of 14 (see below for explanation) to reduce the impact of potential low-quality questionnaire responses. Data cleaning resulted in a final sample set of 19,084 participants.

### 2.2 The human-animal bond questionnaire

The ISPOV measures the human-animal bond across four distinct dimensions: attachment, humanisation, commitment, and integration. Fourteen questions were implemented in the survey, with each question using a 5-point Likert-type response scale (strongly agree, agree, neutral (neither agree nor disagree), disagree, or strongly disagree) in respect of veterinary care issues and (mainly) attachment. To calculate the human-animal bond, which will be referred to as HABSCORE hereafter, the answers to each question were added and summed, resulting in a maximum possible score of 70. The full questionnaire can be found in Supplementary File 1.

### 2.3 Statistical analysis

All statistical analyses were conducted in R (22) wherein potential clusters were identified using mclust (23), which applies a model-based clustering approach based on parameterised finite Gaussian mixture models. This approach was chosen for two reasons. First, this clustering approach allows for the data-driven unsupervised discovery of natural groupings within the data, creating a clearer picture of variances in the bond between owners and their pets. Secondly, initial regression analysis showed weak-moderate explanatory power (highest *R*^2^ = 0.40, Supplementary File 2).

The distribution of the Gaussian mixture models from mclust can vary according to their size, orientation and shape, resulting in 14 possible models that vary across these three dimensions. Initial clustering of participants was conducted on the whole dataset. Input data for the clustering included the participant country, gender, age, and the responses to the 14 individual questions making up the HABSCORE. For this questionnaire, age was split into the following year ranges: 18–24, 25–34, 35–44, 45–54, 55–64, and 65+. A sensitivity analysis was carried out using only HABSCORE questions and age.

Pet type was initially included as a clustering variable. However, initial results showed resultant clusters were strongly tied to pet type (i.e., cat or dog). Clustering was then refined by carrying out the analysis on dog and cat owners separately, given the large sample size (10,876 and 8,208 for dog and cat owners, respectively) to identify differentiating features beyond pet type. Clustering results (across models and number of clusters) were compared using the Bayesian Information Criterion (BIC). The elbow method, a visual inspection of the BIC values to determine smaller improvements in performance (24), was used to choose the optimal model and number of clusters (default setting from one to nine clusters) while also ensuring that clusters contained at least 10% of the cohort.

Clusters were then contrasted across a set of variables including: reason and purpose for having a pet, ratings and importance of veterinary qualities, perceived impact pet has had on mental and physical health, and additional services used for pet. These comparisons were compared using a chi-squared test for factor variables and independent samples t-test for numeric variables. Means between clusters were compared using non-parametric methods (e.g., Mann–Whitney U and Kruskal-Wallis tests). Given the number of variables assessed (*n* = 137), a Bonferroni correction was implemented to reduce the risk of Type 1 Errors for multiple testing resulting in a minimum statistically significant *p-*value of 3.7e-4.

## 3 Results

After data cleaning, 19,084 respondents were included for analysis. Demographic data of the sample is shown in Table 1.

**Table 1:** Demographic data of the survey (*n* = 19,084).

Demographic variable	Overall *n* (%)	Dog owners *n* (%)	Cat owners *n* (%)
Country			
Australia	1,038 (5.5%)	563 (5.2%)	475 (5.8%)
Brazil	2,001 (10.5%)	1,362 (12.5%)	639 (7.8%)
China	1,999 (10.5%)	1,192 (11.0%)	807 (9.8%)
France	1,993 (10.4%)	782 (7.2%)	1,211 (14.8%)
Germany	2,065 (10.8%)	955 (8.8%)	1,110 (13.5%)
Japan	2,001 (10.5%)	998 (9.2%)	1,003 (12.2%)
Mexico	1,990 (10.4%)	1,420 (13.1%)	570 (6.9%)
Spain	2,002 (10.5%)	1,386 (12.7%)	616 (7.5%)
United Kingdom	1,995 (10.5%)	1,025 (9.4%)	970 (11.8%)
United States of America	2,000 (10.5%)	1,193 (11.0%)	807 (9.8%)
Age			
18–24	3,059 (16.0%)	1728 (15.9%)	1,331 (16.2%)
25–34	4,848 (25.4%)	2,872 (26.4%)	1976 (24.1%)
35–44	4,284 (22.4%)	2,471 (22.7%)	181 (22.1%)
45–54	3,323 (17.4%)	1900 (17.5%)	1,423 (17.3%)
55–64	2,280 (11.9%)	1,207 (11.1%)	1,073 (13.1%)
65+	1,290 (6.8%)	698 (6.4%)	592 (7.2%)
Gender			
Female	10,251 (53.7%)	5,751 (52.9%)	4,500 (54.8%)
Male	8,833 (46.3%)	5,125 (47.1%)	3,708 (45.2%)
Pet type			
Dog	10,876 (57.0%)	10,876 (100%)	0 (0%)
Cat	8,208 (43.0%)	0 (0%)	8,208 (100%)

### 3.1 Two types of dog owners identified using HABSCORE data

**Figure 1:**
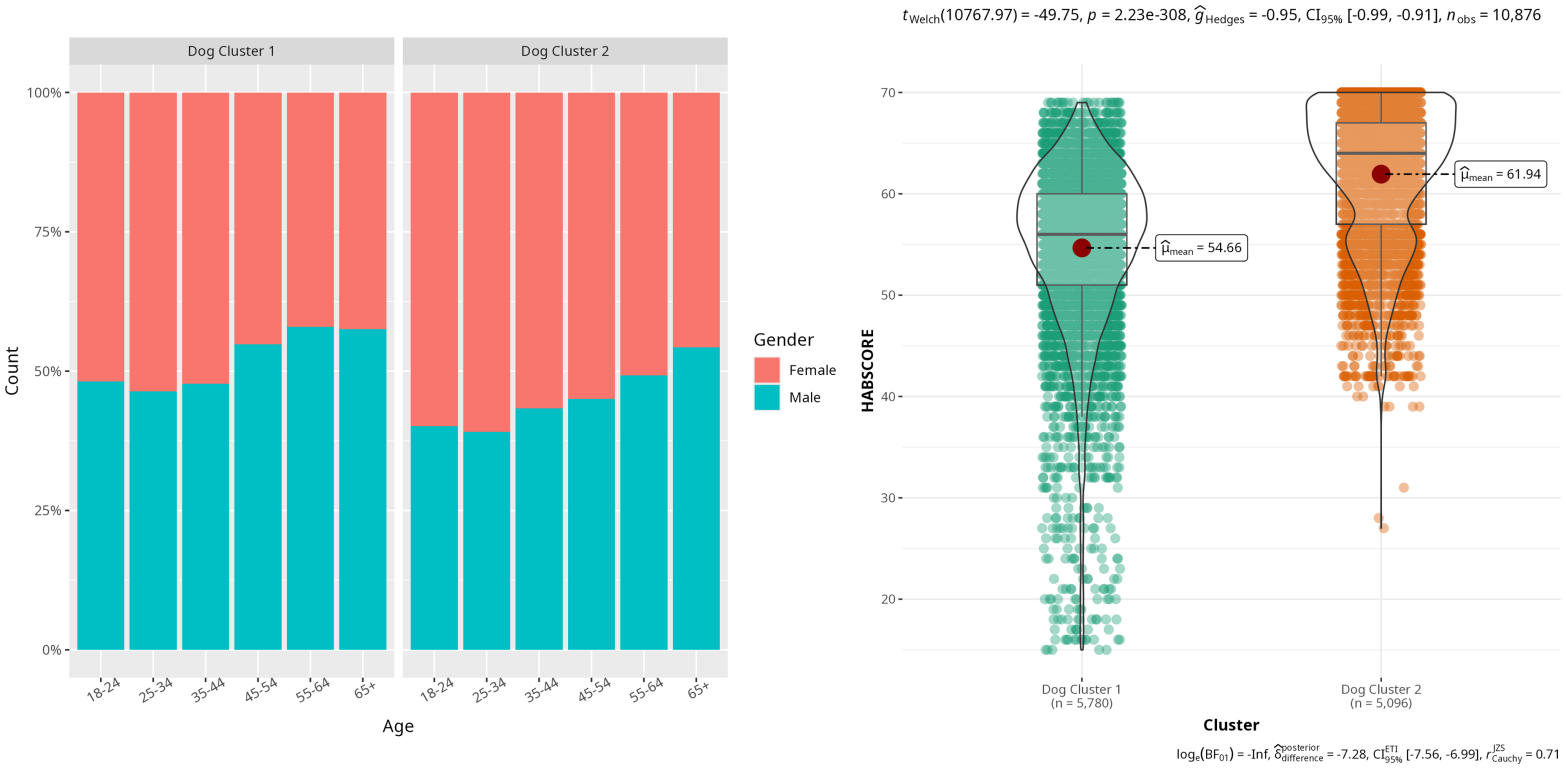
Left – Proportion of gender across age in the two dog clusters; Right – Boxplots of the calculated HABSCORE in the two dog clusters.

In dog owners, two clusters of varying volume, orientation, and shape were identified as the best fit for the data (see Supplementary File 3; Figure 1). These two clusters are defined by a significantly different mean HABSCORE (*p*-value < 0.0001) with Cluster 1 (C1) showing a mean HABSCORE of 54.7 (SD ± 8.5) and Cluster 2 (C2) showing a mean HABSCORE of 62.0 (SD ± 6.8).

The distribution of participant country was significantly different between clusters (*p*-value < 0.0001) (Table 2). The biggest difference in cross-cluster proportion by country are China (7.3% difference), Japan (4.0%), Brazil (3.7%), and Mexico (3.4%). Similarly, the distribution of gender, across the two clusters was found to be significantly different (*p*-value < 0.001), with C1 including similar percentages of males and females (50.3 and 49.7% respectively) and C2 including more females than males (56.4% vs. 43.6%), a significant difference between clusters. The distribution of age was found to be significantly different across clusters (*p*-value = 0.0001), with C1 represented by younger participants compared to C2 (Figure 1).

**Table 2:** Cluster representation of dog owners across variables of interest with *p*-values of chi-squared test shown.

Variable	Cluster 1 n (%)	Cluster 2 *n* (%)	*p*-value
Country			<1.0e-4
Australia	259 (4.5%)	304 (6.0%)	
Brazil	622 (10.8%)	740 (14.5%)	
China	830 (14.4%)	362 (7.1%)	
France	480 (8.3%)	302 (6.0%)	
Germany	438 (7.6%)	517 (10.1%)	
Japan	639 (11.0%)	359 (7.0%)	
Mexico	847 (14.6%)	573 (11.2%)	
Spain	667 (11.5%)	719 (14.1%)	
United Kingdom	463 (8.0%)	562 (11.0%)	
United States of America	535 (9.3%)	658 (12.9%)	
Age			1.0e-4
18–24	960(16.6%)	768 (15.1%)	
25–34	1,575 (27.2%)	1,297 (25.4%)	
35–44	1,340 (23.1%)	1,131 (22.2%)	
45–54	976 (16.9%)	924 (18.1%)	
55–64	592 (10.2%)	615 (12.1%)	
65+	337 (5.8%)	361 (7.1%)	
Gender			< 1.0e-4
Male	2,905 (50.3%)	2,220 (43.6%)	
Female	2,875 (49.7%)	2,876 (56.4%)	
Relationship of pet to owner			<1.0e-4
Companion	801 (13.9%)	432 (8.5%)	
Friend	607 (10.5%)	356 (7.0%)	
Family Member	2,488 (43.0%)	2,986 (40.9%)	
Pet	194 (3.4%)	45 (0.9%)	
Child	1,690 (29.2%)	2,177 (42.7%)	
Pet purpose			< 1.0e-4
Companionship	4,620 (79.9%)	4,209 (82.6%)	
Emotional Support Animal	549 (9,5%)	491 (9.5%)	
Guard dog	272 (4.7%)	191 (3.7%)	
Other (please specify)	259 (4.5%)	165 (3.2%)	
Service Animal	64 (1.1%)	31 (0.6%)	
Keep Mice Out/Pest Control	16 (0.3%)	9 (0.2%)	
Budget spent toward pet			< 1.0e-4
I spend a moderate amount on my pet	3,055 (52.9%)	2,403 (47.2%)	
Money is no object when it comes to my pet	1797 (31.1%)	2097 (41.0%)	
I am budget-conscious	928 (16.1%)	602 (11.8%)	
Use of flea medication			< 1.0e-4
No	1938 (33.5%)	1,275 (25.0%)	
Yes	3,842 (66.5%)	1,275 (25.0%)	
Using nutritional supplements			< 1.0e-4
No	4,342 (75.1%)	3,654 (71.7%)	
Yes	1,438 (24.9%)	1,442 (28.3%)	
Using toothbrush			< 1.0e-4
No	3,822 (66.1%)	3,041 (59.7%)	
Yes	1958 (33.9%)	2055 (40.3%)	
Using dentist services			< 1.0e-4
No	4,933 (85.3%)	4,159 (81.6%)	
Yes	847 (14.7%)	937 (18.4%)	
Using annual check ups			< 1.0e-4
No	2,509 (43.4%)	1,675 (32.9%)	
Yes	3,271 (56.6%)	3,421 (67.1%)	
Using vaccines			< 1.0e-4
No	1,602 (27.7%)	1,131 (22.2%)	
Yes	4,178 (72.3%)	3,965 (77.8%)	
Using grooming services			< 1.0e-4
No	2,987 (51.7%)	2097 (41.1%)	
Yes	2,793 (48.3%)	2,999 (58.9%)	
Using screening services			< 1.0e-4
No	4,916 (85.1%)	4,074 (79.9%)	
Yes	864 (14.9%)	1,022 (20.1%)	
Using none of the services			< 1.0e-4
No	5,550 (96.0%)	4,979 (97.7%)	
Yes	230 (4.0%)	117 (2.3%)	
Having health insurance			0.04
I have never had pet insurance	3,238 (56.0%)	2,894 (56.8%)	
I currently have pet insurance	2025 (35.0%)	1815 (35.6%)	
I used to have pet insurance	517 (8.9%)	387 (7.6%)	
Using monitoring devices			0.002
No	5,274 (91. 2%)	4,733 (92.9%)	
Yes	506 (8.8%)	363 (7.1%)	
Using osteoarthritis medication			0.08
No	5,429 (93.9%)	4,827 (94.7%)	
Yes	351 (6.1%)	269 (5.3%)	
Using medicine for skin-related disorders			0.84
No	5,082 (87.9%)	4,488 (88.1%)	
Yes	698 (12.1%)	608 (11.9%)	
Vet visits per year			< 1.0e-4
Less than once a year	360 (6.2%)	242 (4.7%)	
Once a year	1,413 (24.4%)	1,159 (22.7%)	
Twice a year	1,539 (26.6%)	1,428 (28.0%)	
Three times a year	935 (16.2%)	819 (16.1%)	
Four times a year	717 (12.4%)	612 (12.0%)	
Five times a year or more	661 (11.4%)	735 (14.4%)	
Never	155 (2.7%)	101 (2.0%)	
Pet has positive impact on owner’s health			< 1.0e-4
No	446 (7.7%)	265 (5.2%)	
Unsure	279 (4.8%)	137 (2.7%)	
Yes	5,055 (87.5%)	4,695 (92.1%)	
If pet impacts physical health, mental health, or both			
Both my physical and mental health have improved	1,634 (28.3%)	1970 (38.7%)	
My mental health / emotional wellbeing has improved	2,561 (44.3%)	2,123 (41.7%)	
My physical heath has improved	860 (14.9%)	601 (11.8%)	
NA	725 (12.5%)	402 (7.9%)	
Pet helps improve activity levels			< 1.0e-4
No	3,295 (57.0%)	2,384 (46.8%)	
Yes	2,485 (43.0%)	2,712 (53.2%)	
Pet helps provide comfort when owner is sad			< 1.0e-4
No	3,223 (55.8%)	2007 (39.4%)	
Yes	2,557 (44.2%)	3,089 (60.6%)	
Pet helps calm owner when stressed			< 1.0e-4
No	3,479 (60.2%)	2,376 (46.6%)	
Yes	2,301 (39.8%)	2,720 (53.4%)	
Pet helps make owner happy			< 1.0e-4
No	2,244 (38.8%)	1,232 (24.2%)	
Yes	3,536 (61.2%)	3,864 (75.8%)	
Pet helps provide a greater sense of community			< 1.0e-4
No	4,618 (79.9%)	3,843 (75.4%)	
Yes	1,162 (20.1%)	1,253 (24.6%)	
Pet helps decrease owner’s loneliness			< 1.0e-4
No	3,309 (57.2%)	2,428 (47.6%)	
Yes	2,471 (42.8%)	2,668 (52.4%)	
Pet helps provide a sense of purpose			< 1.0e-4
No	3,725 (64.4%)	2,553 (50.1%)	
Yes	2055 (35.6%)	2,543 (49.9%)	
Pet adds to owner’s happiness			< 1.0e-4
No	2,562 (44.3%)	1,438 (28.2%)	
Yes	3,218 (55.7%)	3,658 (71.8%)	
Pet has improved owner’s self-rating of health			< 1.0e-4
No	3,769 (65.2%)	2,554 (50.1%)	
Yes	2011 (34.8%)	2,542 (49.9%)	
Medical doctor’s opinion that pet has improved health			0.09
No	5,291 (91.5%)	4,617 (90.6%)	
Yes	489 (8.5%)	479 (9.4%)	
Pet provides no help			< 1.0e-4
No	5,684 (98.3%)	5,058 (99.3%)	
Yes	96 (1.7%)	38 (0.7%)	
Importance of veterinarian knowledge			< 1.0e-4
Very important	3,269 (56.6%)	3,790 (74.4%)	
Important	1,689 (29.2%)	929 (18.2%)	
Moderately important	539 (9.3%)	275 (5.4%)	
Slightly important	232 (4.0%)	80 (1.6%)	
Not at all important	51 (0.9%)	22 (0.4%)	
Importance of veterinarian bond with pet			< 1.0e-4
Very important	2,176 (37.6%)	3,093 (60.7%)	
Important	2,274 (39.3%)	1,355 (26.6%)	
Moderately important	897 (15.5%)	487 (9.6%)	
Slightly important	343 (5.9%)	128 (2.5%)	
Not at all important	90 (1.6%)	33 (0.6%)	
Importance of veterinarian price			< 1.0e-4
Very important	2036 (35.2%)	2,340 (45.9%)	
Important	2,248 (38.9%)	1,658 (32.5%)	
Moderately important	1,071 (18.5%)	765 (15.0%)	
Slightly important	360 (6.2%)	252 (4.9%)	
Not at all important	65 (1.1%)	81 (1.6%)	
Importance of veterinarian empathy toward pet			< 1.0e-4
Very important	2,768 (47.9%)	3,556 (69.8%)	
Important	1998 (34.6%)	1,082 (21.2%)	
Moderately important	674 (11.7%)	326 (6.4%)	
Slightly important	287 (5.0%)	110 (2.2%)	
Not at all important	53 (0.9%)	22 (0.4%)	
Importance of veterinarian explanations			< 1.0e-4
Very important	3,035 (52.5%)	3,534 (69.3%)	
Important	1991 (34.4%)	1,176 (23.1%)	
Moderately important	513 (8.9%)	286 (5.6%)	
Slightly important	196 (3.4%)	81 (1.6%)	
Not at all important	45 (0.8%)	19 (0.4%)	
Importance of veterinarian empathy toward owner			< 1.0e-4
Very important	2,160 (37.4%)	2,945 (57.8%)	
Important	2,270 (39.3%)	1,433 (28.1%)	
Moderately important	920 (15.9%)	531 (10.4%)	
Slightly important	345 (6.0%)	151 (3.0%)	
Not at all important	85 (1.5%)	36 (0.7%)	
Importance of veterinarian incorporating owner decisions			< 1.0e-4
Very important	2,424 (41.9%)	3,141 (61.6%)	
Important	2,197 (38.0%)	1,392 (27.3%)	
Moderately important	780 (13.5%)	424 (8.3%)	
Slightly important	327 (5.7%)	121 (2.4%)	
Not at all important	52 (0.9%)	18 (0.4%)	
Importance of veterinarian staff knowledge			< 1.0e-4
Very important	2,745 (47.5%)	3,422 (67.2%)	
Important	2,143 (37.1%)	1,233 (24.2%)	
Moderately important	589 (10.2%)	315 (6.2%)	
Slightly important	248 (4.3%)	111 (2.2%)	
Not at all important	55 (1.0%)	15 (0.3%)	
Importance of veterinarian environment			< 1.0e-4
Very important	2,309 (39.9%)	3,111 (61.0%)	
Important	2,355 (40.7%)	1,431 (28.1%)	
Moderately important	775 (13.4%)	400 (7.8%)	
Slightly important	300 (5.2%)	135 (2.6%)	
Not at all important	41 (0.7%)	19 (0.4%)	
Rating of veterinarian knowledge			< 1.0e-4
Excellent	2,674 (46.3%)	3,207 (62.9%)	
Good	2,364 (40.9%)	1,473 (28.9%)	
Average	546 (9.4%)	315 (6.2%)	
Poor	45 (0.8%)	13 (0.3%)	
Very poor	10 (0.2%)	3 (0.1%)	
NA	141 (2.4%)	85 (1.7%)	
Rating of veterinarian bond with pet			< 1.0e-4
Excellent	2055 (35.6%)	2,763 (54.2%)	
Good	2,668 (46.2%)	1763 (34.6%)	
Average	837 (14.5%)	447 (8.8%)	
Poor	62 (1.1%)	33 (0.6%)	
Very poor	17 (0.3%)	5 (0.1%)	
NA	141 (2.4%)	85 (1.7%)	
Rating of veterinarian price			< 1.0e-4
Excellent	1,450 (25.1%)	1792 (35.2%)	
Good	2,506 (43.4%)	1918 (37.6%)	
Average	1,456 (25.2%)	1,140 (22.4%)	
Poor	185 (3.2%)	123 (2.4%)	
Very poor	42 (0.7%)	38 (0.7%)	
NA	141 (2.4%)	85 (1.7%)	
Rating of veterinarian empathy toward pet			< 1.0e-4
Excellent	2,331 (40.3%)	2,934 (57.6%)	
Good	2,524 (43.7%)	1,666 (32.7%)	
Average	721 (12.5%)	380 (7.5%)	
Poor	50 (0.9%)	27 (0.5%)	
Very poor	13 (0.2%)	4 (0.1%)	
NA	141 (2.4%)	85 (1.7%)	
Rating of veterinarian explanations			< 1.0e-4
Excellent	2,516 (43.5%)	3,010 (59.1%)	
Good	2,502 (43.3%)	1,659 (32.6%)	
Average	556 (9.6%)	323 (6.3%)	
Poor	54 (0.9%)	15 (0.3%)	
Very poor	11 (0.2%)	4 (0.1%)	
NA	141 (2.4%)	85 (1.7%)	
Rating of veterinarian empathy toward owner			<1.0e-4
Excellent	2076 (35.9%)	2,718 (53.3%)	
Good	2,697 (46.7%)	1804 (35.4%)	
Average	798 (13.8%)	454 (8.9%)	
Poor	56 (1.0%)	27 (0.5%)	
Very poor	12 (0.2%)	8 (0.2%)	
NA	141 (2.4%)	85 (1.7%)	
Rating of veterinarian incorporating owner decisions			< 1.0e-4
Excellent	2,277 (39.4%)	2,885 (56.6%)	
Good	2,557 (44.2%)	1700 (33.4%)	
Average	719 (12.4%)	396 (7.8%)	
Poor	73 (1.3%)	22 (0.4%)	
Very poor	13 (0.2%)	8 (0.2%)	
NA	141 (2.4%)	85 (1.7%)	
Rating of veterinarian staff			<1.0e-4
Excellent	2,205 (38.1%)	2,782 (54.6%)	
Good	2,595 (44.9%)	1775 (34.8%)	
Average	769 (13.3%)	413 (8.1%)	
Poor	55 (1.0%)	35 (0.7%)	
Very poor	15 (0.3%)	6 (0.1%)	
NA	141 (2.4%)	85 (1.7%)	
Rating of veterinarian environment			< 1.0e-4
Excellent	2070 (35.8%)	2,646 (51.9%)	
Good	2,707 (46.8%)	1852 (36.3%)	
Average	771 (13.3%)	473 (9.3%)	
Poor	86 (1.5%)	35 (0.7%)	
Very poor	5 (0.1%)	5 (0.1%)	

For brevity variables presented are a subset of all tested variables.

### 3.2 Relationship to companion animal and their purpose

**Figure 2:**
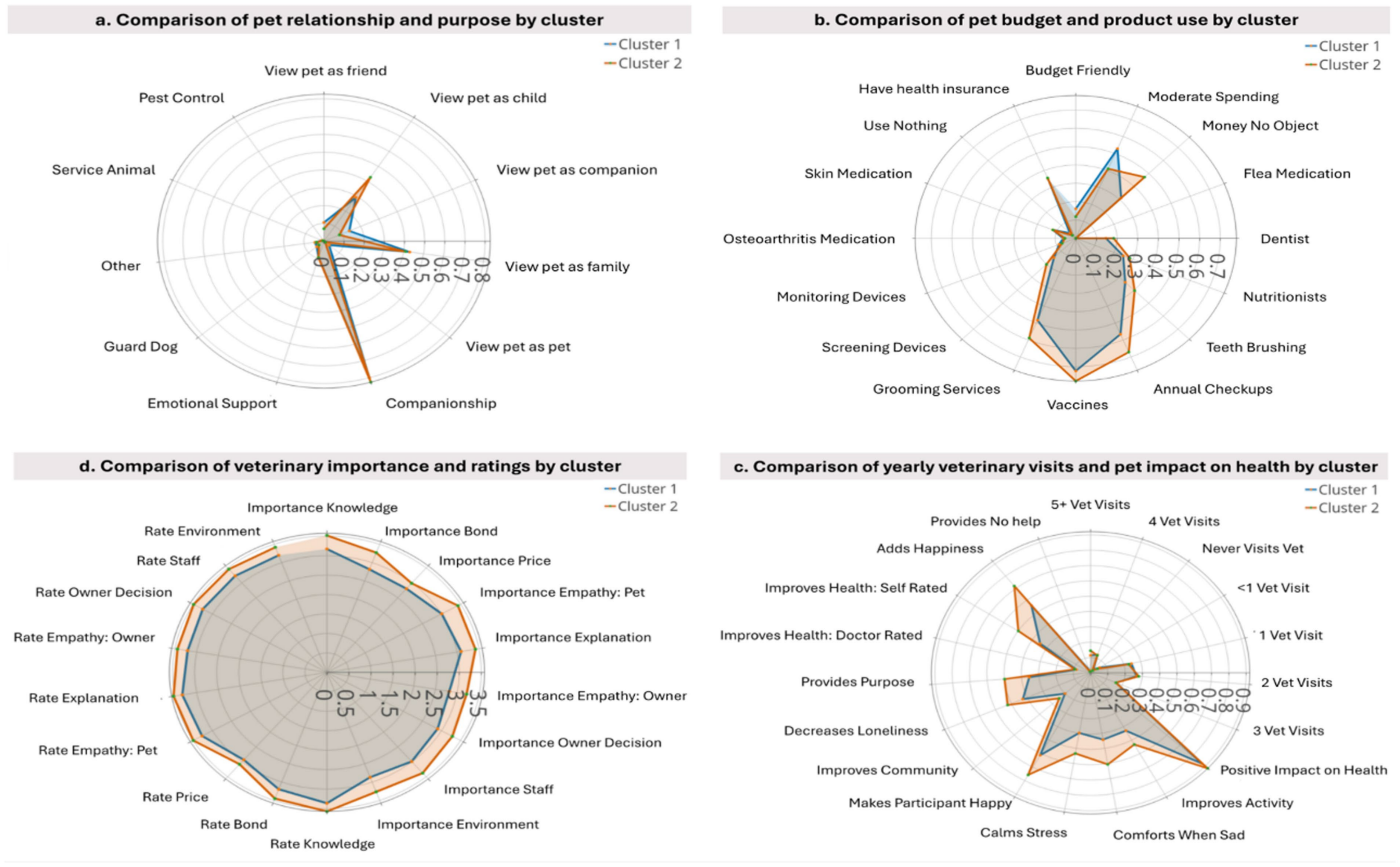
Radar plots that show clusters of dog owners based on aspects of the HABRI questionnaire. **(a)** Clusters based on pet relationship to owner and pet purpose; **(b)** clusters based on budget and product use for pet; **(c)** clusters showing importance of veterinary qualities and rating of current veterinarian on those qualities; **(d)** clusters based on rate of veterinary visits and pet impact on owner health.

Differences between the two clusters of dog owners on the relationship to pet are summarized in Figure 2 and Table 2. How participants think or feel about their dog showed significant differences in distribution between clusters. C2 was more likely to view their dog as child (42.7% vs. 29.2% in C1), while C1 showed a larger trend to viewing their dog as a companion (13.9% vs. 8.5% in C2), a friend (10.5% vs. 7.0%), or just a pet (3.4% vs. 0.9%). The reason for participants having a dog likewise showed a significant difference in distribution, with C1 showcasing a larger shift toward having a dog for work-related reasons including guard dog (4.7% vs. 3.2% in C2), pest control (0.3% vs. 0.2%), and service animal (1.1% vs. 0.6%). The average response to all these questions also differed significantly between clusters with C2 showing a higher average for perceived relationship (4.16, 3.81 in C1) suggesting a greater tendency toward feeling their dog is like a child and a lower average pet purpose (1.35, 1.43 in C1), signifying a tendency toward companionship as purpose for their dog. Note all results comparing averages between clusters for dog and cat owners can be seen in Supplementary File 4, Table 1.

### 3.3 Budget and pet product use

The perceived care budget significantly differed between clusters (Table 2). Participants in C2 had a higher rate of response to “money is no object” (41.0%) compared to C1 (31.1%) when it comes to their dog, while C1 was more represented as budget-conscious (16.1% vs. 11.8% for C1 and C2, respectively), and spending a moderate amount of money on their dog. Additionally, the average response on pet budget between C1 (2.15) and C2 (2.35) significantly differed between clusters.

C2 pet owners group showed a higher response compared to C1 for: using flea medication (75.0% vs. 66.5%), using nutrition supplements (28.3% vs. 24.9%), using tooth brush (40.3% vs. 33.9%), using dentist services (18.4% vs. 14.7%), annual check-ups (67.1% vs. 56.6%), using vaccines (77.8% vs. 72.3%), use grooming services (58.9% vs. 48.3%), and the use of screening services (20.1% vs. 14.9%). C1 had a higher response compared to C2 for annual check-ups (43.4% vs. 32.9%), vaccination (27.7% vs. 22.2%) and grooming (48.3% vs. 41.1%). Overall, 97.7% of C2 used the above-mentioned services, compared to 96.0% of the C1 pet owners cluster group. Significant differences in the average responses between clusters were also present in the above variables.

When considering the average response, significant differences were seen across almost all variables. In almost all other variables C2 had a higher average response signifying greater likelihood of reporting they use these services. The exception to this is “using none of these services” where the lower average response by C2 signifies a lower likelihood of selecting yes to this question.

No significant differences existed between clusters in having health insurance for their dog, using monitoring devices, medicine for osteoarthritis, and medicine for skin-related disorders in either the distribution of responses or average score.

### 3.4 Important veterinary qualities and perceived rating of current veterinarian

Percentage differences between clusters were also present when participants were asked about the importance of a variety of veterinary factors and to then rate their own veterinarians on these factors (Table 2). C2 consistently showed higher ratings across all factors, suggesting greater importance for these qualities in their veterinary choices and rating their own veterinarians higher than C1. For example, C2 was more likely to rate the following aspects as very important compared to C1: the importance of bond between veterinarian and dog (60.7% vs. 37.6% respectively), empathy toward pet (69.8% vs. 47.9%), empathy toward owner (57.8% vs. 37.4%), and veterinarian knowledge (74.4% vs. 56.6%). This was also seen across participant ratings of their veterinarians with C2 rating their veterinarian as “excellent” at higher rate than C1 across: bond with vet (54.2% vs. 35.6% in C1), empathy toward dog (57.6% vs. 40.3%), empathy toward owner (53.3% vs. 35.9%), and veterinarian knowledge (62.9% vs. 46.3%). Potentially paradoxically, C2 was also more likely to say the price of their veterinarian as “very important” at a higher rate (45.9% in C2 vs. 35.2% in C1) when they may consider money no object for their dog.

The average response differed significantly between both clusters, with C2 showing higher average scores than C1 across all variables. This shows that not only do members of C2 gage certain veterinarian qualities as more important than C1 members on average, but they are also more likely to rate their veterinarians more highly.

The two clusters significantly differed relative to yearly vet visits, with C2 showing a greater prevalence for five or more veterinary visits per year (14.4% vs. 11.4% in C1). The average response was significantly higher C2, showing a propensity to bring their dogs into the veterinarian more often than C1.

### 3.5 Perceived impact on owner’s health

In respect of the dogs’ impact on participants’ health, C2 tended to state that their dogs had a positive impact on a variety of health-related factors including physical activity (53.2% vs. 43.0%), stress (53.4% vs. 39.8%), happiness (75.8% vs. 61.2%), and loneliness (52.4% vs. 42.8% in C1). Overall, differences on the responses between C1 and C2 regarding the impact of a dog on pet owner’s health were statistically significant across all categories except for “Being told by a medical doctor that the participant’s pet has improved their health” (Table 2). The average responses were likewise significantly higher for C2 than C1 showing that they were more likely to report their dogs helping across these health-related factors than C1.

### 3.6 Cat owners are clustered into 3 types

**Figure 3:**
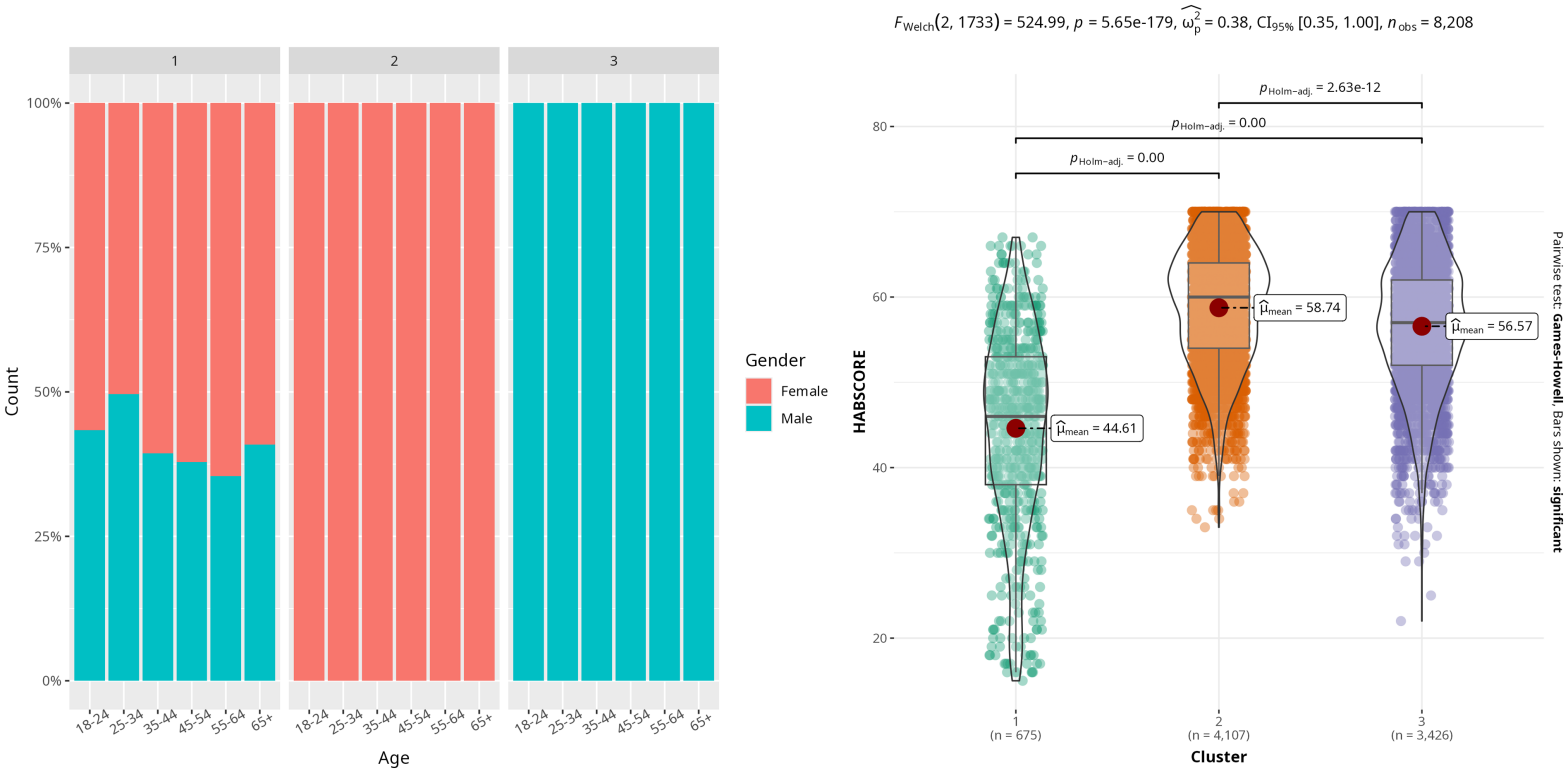
Left – Proportion of gender of companion animal guardian with respect to age categories in the three cat clusters identified in this study; Right – Boxplots of the calculated HABSCORE in the three cat owner clusters.

In the case of cat owners, three clusters of varying volume, orientation, and shape were identified as the best fit for the data (see Supplementary File 3; Figure 2). These three clusters display a significantly different mean HABSCORE (*p*-value < 1e-4) with Cat owner Cluster 1 (CC1) showing a mean HABSCORE of 44.6 (± 11.4), Cat owner Cluster 2 (CC2) showing a mean HABSCORE of 58.7 (SD ± 6.8), and Cat owner Cluster 3 (CC3) mean of 56.6 (SD ± 7.6) (Figure 3). The distribution of participant country was significantly different than might be expected between clusters (*p*-value < 1e-4), with CC1 showing a stronger distribution of western countries, noting that Japan sits as an exception to this pattern. Gender distribution across the three clusters was found to be significantly different than expected (*χ*^2^ = 7545.1, *p*-value < 1e-4). Gender representation was roughly equal for CC1 (female: 58.2%, male: 41.8%), but CC2 was all female (4,107) and CC3 was all male (3,426). Lastly, the distribution of age was found to be significantly different across clusters (*p*-value < 1e-4), with CC1 tending to include younger participants, and CC3 tending to include older participants (Figure 3).

Significant differences in the average age responses were seen between CC2 and CC3 as well as between all cat cluster comparisons for the average response to gender.

### 3.7 Relationship of cat owners to their pets

**Figure 4:**
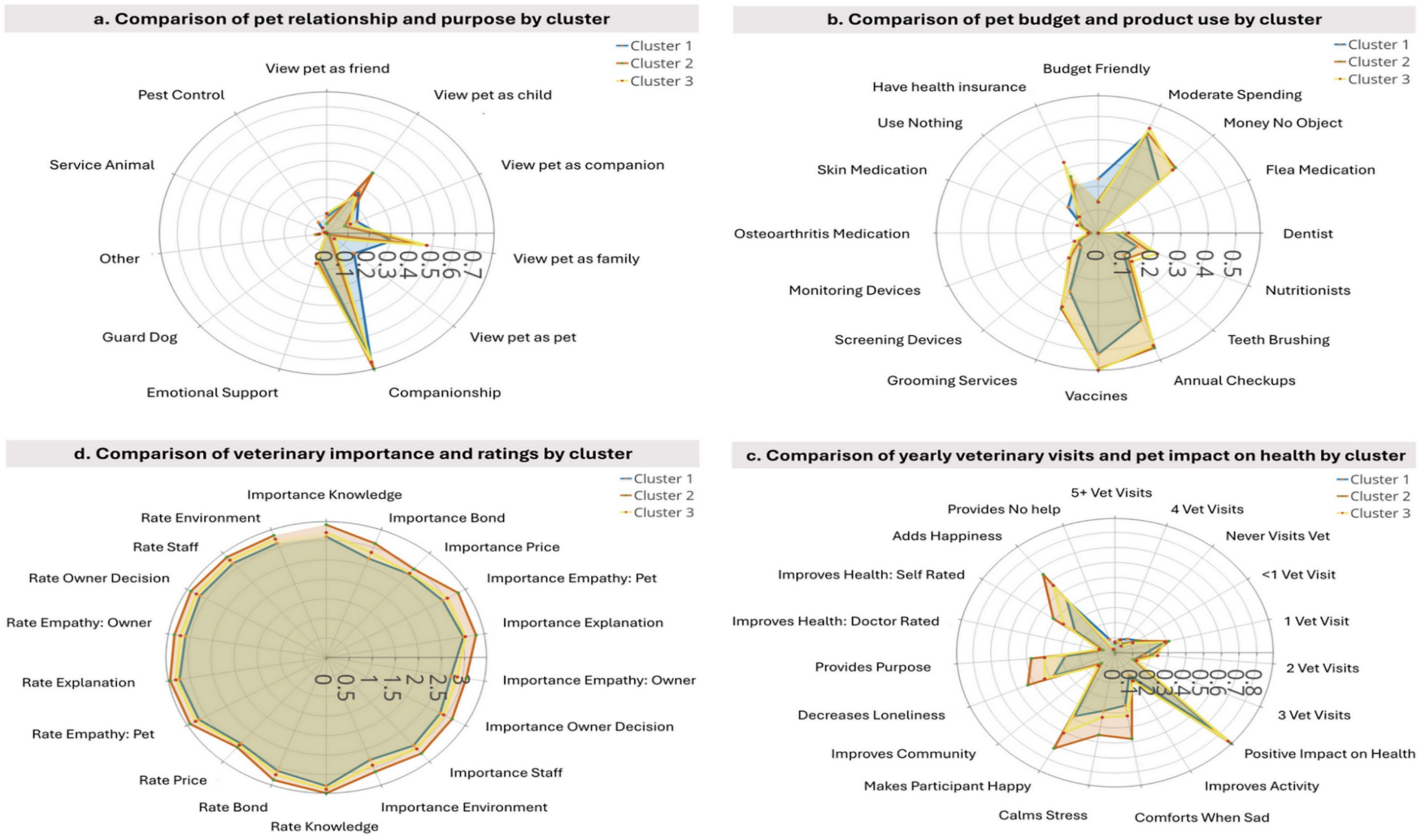
Radar plots comparing clusters of cat owners based on aspects of the HABRI questionnaire. Panel **(a)** shows data on cat relationship to owner and cat purpose; **(b)** on budget and product use for cat; **(c)** on rating importance of veterinary qualities and rating of current veterinarian on those qualities; **(d)** on comparison of yearly veterinary visits and cat impact on owner health.

Results of the cat owner characteristics across the three clusters are summarized in Figure 4 and Table 3. The three cat clusters significantly differed on their perception they have of their cats (*χ*^2^ = 615.29, *p*-value <1e-4). More precisely, the CC2 viewed their cat like a child (39.6% in CC2 vs. 27.9% in CC1 and 25.0% in CC3), while participants in CC1 and CC3 were more likely to view their cat as a pet (17.0 and 4.7%, respectively, vs. 1.5% in CC2), companion (15.6 and 12.1%, respectively, vs. 9.3% in CC2), or friend (8.9 and 10.9%, respectively, vs. 5.3% in CC2) (Figure 4). Participants in CC1 were less likely to view their cat as a family member (30.7% vs. 44.3% in CC2 and 47.3% in CC3). Similarly, the pet purpose perception of the three cat clusters was significantly different (*χ*^2^ = 137.55, *p*-value <1e-4). CC3 was more likely to report getting a cat for emotional support (17.5% vs. 13% in CC1 and 15.7% in CC2), while CC2 was more likely to get a cat for companionship (78.3% vs. 73.3% in CC1 and 74.3% in CC3), and participants in CC1 were more likely to get a cat for pest control (7.3% vs. 0.9% in CC2 and 3.5% in CC3). Similarly, significant differences on “money spent on their cat” were observed (*χ*^2^ = 53.65, *p*-value < 1e-4). In CC2a higher percentage responded as “money is no object” when it comes to their cat (39.6% vs. 31.3% in CC1 and 38.2% in CC3), while CC1 was more likely to report being budget conscious (23.3% vs. 13.9% in CC2 and 13.2% in CC3); C3 on the other hand was more likely to report spending a moderate amount on their cat (38.6% vs. 45.5% in CC1 and 48.6% in CC2). cat (38.6% vs. 45.5% in CC1 and 48.6% in CC2). When assessing the average response to relationship of the owner to their, significant differences were present across all cat clusters. CC2 (mean 4.11) showed the highest average compared to CC1 (3.37) and CC3 (3.76) suggesting a stronger view of their relationship.

**Table 3:** Cluster representation of cat owners across variables of interest with p-values of chi-squared test.

Variable	Cluster 1 *n* (%)	Cluster 2 *n* (%)	Cluster 3 *n* (%)	*p*-value
Country				<1.0e-4
Australia	21 (3.1%)	238 (5.8%)	216 (6.3%)	
Brazil	20 (3.0%)	346 (8.4%)	273 (8.0%)	
China	19 (2.8%)	483 (11.8%)	305 (8.9%)	
France	138 (20.4%)	614 (15.0%)	459 (13.4%)	
Germany	89 (13.2%)	592 (14.4%)	429 (12.5%)	
Japan	78 (11.6%)	375 (9.1%)	550 (16.1%)	
Mexico	136 (20.1%)	205 (5.0%)	229 (6.7%)	
Spain	48 (7.1%)	317 (7.7%)	251 (7.3%)	
United Kingdom	68 (10.1%)	495 (12.1%)	407 (11.9%)	
United States of America	58 (8.6%)	442 (10.8%)	307 (9.0%)	
Age				<1.0e-4
18–24	159 (23.6%)	657 (16.0%)	515 (15.0%)	
25–34	141 (20.9%)	1,067 (26.0%)	768 (22.4%)	
35–44	132 (19.6%)	910 (22.2%)	771 (22.5%)	
45–54	103 (15.3%)	680 (16.6%)	640 (18.7%)	
55–64	96 (14.2%)	529 (12.9%)	448 (13.1%)	
65+	44 (6.5%)	264 (6.4%)	284 (8.3%)	
Gender				< 1.0e-4
Male	282 (41.8%)		3,426 (100.0%)	
Female	393 (58.2%)	4,107 (100.0%)		
Relationship of pet to owner				<1.0e-4
Companion	105 (15.6%)	383 (9.3%)	415 (12.1%)	
Friend	60 (8.9%)	216 (5.3%)	375 (10.9%)	
Family Member	207 (30.7%)	1819 (44.3%)	1,621 (47.3%)	
Pet	115 (17.0%)	61 (1.5%)	160 (4.7%)	
Child	188 (27.9%)	1,628 (39.6%)	855 (25.0%)	
Pet purpose				< 1.0e-4
Companionship	495 (73.3%)	3,216 (78.3%)	2,547 (74.3%)	
Emotional Support Animal	88 (13.0%)	645 (15.7%)	601 (17.5%)	
Other (please specify)	36 (5.3%)	175 (4.3%)	119 (3.5%)	
Service Animal	7 (1.0%)	34 (0.8%)	38 (1.1%)	
Keep Mice Out/Pest Control	49 (7.3%)	37 (0.9%)	121 (3.5%)	
Budget spent toward pet				< 1.0e-4
I spend a moderate amount on my pet	307 (45.5%)	1913 (46.6%)	1,665 (48.6%)	
Money is no object when it comes to my pet	211 (31.3%)	1,625 (39.6%)	1,308 (38.2%)	
I am budget-conscious	157 (23.3%)	569 (13.9%)	453 (13.2%)	
Use of flea medication				< 1.0e-4
No	329 (48.7%)	1,604 (39.1%)	1,468 (42.8%)	
Yes	346 (51.3%)	2,503 (60.9%)	1958 (57.2%)	
Using nutritional supplements				< 1.0e-4
No	573 (84.9%)	3,292 (80.2%)	2,630 (76.8%)	
Yes	102 (15.1%)	815 (19.8%)	796 (23.2%)	
Using toothbrush				0.01
No	585 (86.7%)	3,478 (84.7%)	2,834 (82.7%)	
Yes	90 (13.3%)	629 (15.3%)	592 (17.3%)	
Using dentist services				1.10e-4
No	633 (93.8%)	3,739 (91.0%)	3,050 (89.0%)	
Yes	42 (6.2%)	368 (9.0%)	376 (11.0%)	
Using annual check ups				< 1.0e-4
No	401 (59.4%)	1918 (46.7%)	1,637 (47.8%)	
Yes	274 (40.6%)	2,189 (53.3%)	1789 (52.2%)	
Using vaccines				0.003
No	325 (48.1%)	1712 (41.7%)	1,410 (41.2%)	
Yes	350 (51.9%)	2,395 (58.3%)	2016 (58.8%)	
Using grooming services				2.50e-4
No	492 (72.9%)	2,665 (64.9%)	2,252 (65.7%)	
Yes	183 (27.1%)	1,442 (35.1%)	1,174 (34.3%)	
Using screening services				1.10e-4
No	615 (91.1%)	3,519 (85.7%)	2,907 (84.9%)	
Yes	60 (8.9%)	588 (14.3%)	519 (15.1%)	
Using none of the services				< 1.0e-4
No	570 (84.4%)	3,683 (89.7%)	3,094 (90.3%)	
Yes	105 (15.6%)	424 (10.3%)	332 (9.7%)	
Having health insurance				0.04
I have never had pet insurance	459 (68.0%)	2,773 (67.5%)	2003 (58.5%)	
I currently have pet insurance	150 (22.2%)	1,076 (26.2%)	1,123 (32.8%)	
I used to have pet insurance	66 (9.8%)	258 (6.3%)	300 (8.8%)	
Using monitoring devices				6.60e-4
No	623 (92.3%)	3,824 (93.1%)	3,108 (90.7%)	
Yes	52 (7.7%)	283 (6.9%)	318 (9.3%)	
Using osteoarthritis medication				0.09
No	657 (97.3%)	3,989 (97.1%)	3,299 (96.3%)	
Yes	18 (2.7%)	118 (2.9%)	127 (3.7%)	
Using medicine for skin-related disorders				0.01
No	636 (94.2%)	3,830 (93.3%)	3,142 (91.7%)	
Yes	39 (5.8%)	277 (6.7%)	284 (8.3%)	
Vet visits per year				< 1.0e-4
Less than once a year	101 (15.0%)	593 (14.4%)	411 (12.0%)	
Once a year	196 (29.0%)	1,284 (31.3%)	993 (29.0%)	
Twice a year	109 (16.1%)	861 (21.0%)	825 (24.1%)	
Three times a year	78 (11.6%)	470 (11.4%)	456 (13.3%)	
Four times a year	63 (9.3%)	360 (8.8%)	309 (9.0%)	
Five times a year or more	50 (7.4%)	245 (6.0%)	238 (6.9%)	
Never	78 (11.6%)	294 (7.2%)	194 (5.7%)	
Pet has positive impact on owner’s health				< 1.0e-4
No	98 (14.5%)	260 (6.3%)	265 (7.7%)	
Unsure	70 (10.4%)	184 (4.5%)	192 (5.6%)	
Yes	507 (75.1%)	3,663 (89.2%)	2,969 (86.7%)	
If pet impacts physical health, mental health, or both				
Both my physical and mental health have improved	109 (16.1%)	899 (21.9%)	711 (20.8%)	
My mental health / emotional wellbeing has improved	333 (49.3%)	2,456 (59.8%)	1819 (53.1%)	
My physical heath has improved	65 (9.6%)	308 (7.5%)	439 (12.8%)	
NA	168 (24.9%)	444 (10.8%)	457 (13.3%)	
Pet helps improve activity levels				0.02
No	561 (83.1%)	3,284 (80.0%)	2,693 (78.6%)	
Yes	114 (16.9%)	823 (20.0%)	733 (21.4%)	
Pet helps provide comfort when owner is sad				< 1.0e-4
No	435 (64.4%)	1723 (42.0%)	1967 (57.4%)	
Yes	240 (35.6%)	2,384 (58.0%)	1,459 (42.6%)	
Pet helps calm owner when stressed				< 1.0e-4
No	411 (60.9%)	1834 (44.7%)	1933 (56.4%)	
Yes	264 (39.1%)	2,273 (55.3%)	1,493 (43.6%)	
Pet helps make owner happy				< 1.0e-4
No	351 (52.0%)	1,137 (27.7%)	1,346 (39.3%)	
Yes	324 (48.0%)	2,970 (72.3%)	2080 (60.7%)	
Pet helps provide a greater sense of community				0.003
No	601 (89.0%)	3,687 (89.8%)	2,990 (87.3%)	
Yes	74 (11.0%)	420 (10.2%)	436 (12.7%)	
Pet helps decrease owner’s loneliness				< 1.0e-4
No	422 (62.5%)	1907 (46.4%)	1945 (56.8%)	
Yes	253 (37.5%)	2,200 (53.6%)	1,481 (43.2%)	
Pet helps provide a sense of purpose				< 1.0e-4
No	486 (72.0%)	2,162 (52.6%)	2063 (60.2%)	
Yes	189 (28.0%)	1945 (47.4%)	1,363 (39.8%)	
Pet adds to owner’s happiness				< 1.0e-4
No	377 (55.9%)	1,401 (34.1%)	1,490 (43.5%)	
Yes	298 (44.1%)	2,706 (65.9%)	1936 (56.5%)	
Pet has improved owner’s self-rating of health				< 1.0e-4
No	493 (73.0%)	2,404 (58.5%)	2,238 (65.3%)	
Yes	182 (27.0%)	1703 (41.5%)	1,188 (34.7%)	
Medical doctor’s opinion that pet has improved health				7.90e-4
No	629 (93.2%)	3,821 (93.0%)	3,110 (90.8%)	
Yes	46 (6.8%)	286 (7.0%)	316 (9.2%)	
Pet provides no help				< 1.0e-4
No	612 (90.7%)	4,047 (98.5%)	3,341 (97.5%)	
Yes	63 (9.3%)	60 (1.5%)	85 (2.5%)	
Importance of veterinarian knowledge				< 1.0e-4
Very important	365 (54.1%)	2,819 (68.6%)	1979 (57.8%)	
Important	197 (29.2%)	960 (23.4%)	1,011 (29.5%)	
Moderately important	68 (10.1%)	223 (5.4%)	300 (8.8%)	
Slightly important	31 (4.6%)	88 (2.1%)	110 (3.2%)	
Not at all important	14 (2.1%)	17 (0.4%)	26 (0.8%)	
Importance of veterinarian bond with pet				< 1.0e-4
Very important	245 (36.3%)	2,139 (52.1%)	1,339 (39.1%)	
Important	206 (30.5%)	1,339 (32.6%)	1,301 (38.0%)	
Moderately important	122 (18.1%)	456 (11.1%)	550 (16.1%)	
Slightly important	60 (8.9%)	144 (3.5%)	187 (5.5%)	
Not at all important	42 (6.2%)	29 (0.7%)	49 (1.4%)	
Importance of veterinarian price				< 1.0e-4
Very important	248 (36.7%)	1763 (42.9%)	1,218 (35.6%)	
Important	227 (33.6%)	1,505 (36.6%)	1,310 (38.2%)	
Moderately important	129 (19.1%)	633 (15.4%)	655 (19.1%)	
Slightly important	52 (7.7%)	163 (4.0%)	186 (5.4%)	
Not at all important	19 (2.8%)	43 (1.0%)	57 (1.7%)	
Importance of veterinarian empathy toward pet				< 1.0e-4
Very important	304 (45.0%)	2,598 (63.3%)	1,674 (48.9%)	
Important	199 (29.5%)	1,047 (25.5%)	1,182 (34.5%)	
Moderately important	102 (15.1%)	321 (7.8%)	395 (11.5%)	
Slightly important	48 (7.1%)	121 (2.9%)	150 (4.4%)	
Not at all important	22 (3.3%)	20 (0.5%)	25 (0.7%)	
Importance of veterinarian explanations				< 1.0e-4
Very important	342 (50.7%)	2,622 (63.8%)	1753 (51.2%)	
Important	209 (31.0%)	1,150 (28.0%)	1,196 (34.9%)	
Moderately important	73 (10.8%)	223 (5.4%)	320 (9.3%)	
Slightly important	33 (4.9%)	87 (2.1%)	129 (3.8%)	
Not at all important	18 (2.7%)	25 (0.6%)	28 (0.8%)	
Importance of veterinarian empathy toward owner				< 1.0e-4
Very important	252 (37.3%)	2,129 (51.8%)	1,368 (39.9%)	
Important	202 (29.9%)	1,336 (32.5%)	1,240 (36.2%)	
Moderately important	128 (19.0%)	434 (10.6%)	584 (17.0%)	
Slightly important	60 (8.9%)	170 (4.1%)	182 (5.3%)	
Not at all important	33 (4.9%)	38 (0.9%)	52 (1.5%)	
Importance of veterinarian incorporating owner decisions				< 1.0e-4
Important	226 (33.5%)	1,327 (32.3%)	1,250 (36.5%)	
Very important	292 (43.3%)	2,267 (55.2%)	1,506 (44.0%)	
Moderately important	83 (12.3%)	368 (9.0%)	460 (13.4%)	
Slightly important	58 (8.6%)	122 (3.0%)	174 (5.1%)	
Not at all important	16 (2.4%)	23 (0.6%)	36 (1.1%)	
Importance of veterinarian staff knowledge				< 1.0e-4
Important	217 (32.1%)	1,272 (31.0%)	1,200 (35.0%)	
Very important	313 (46.4%)	2,410 (58.7%)	1709 (49.9%)	
Moderately important	87 (12.9%)	298 (7.3%)	372 (10.9%)	
Slightly important	41 (6.1%)	107 (2.6%)	121 (3.5%)	
Not at all important	17 (2.5%)	20 (0.5%)	24 (0.7%)	
Importance of veterinarian environment				< 1.0e-4
Very important	264 (39.1%)	2,175 (53.0%)	1,448 (42.3%)	
Important	240 (35.6%)	1,406 (34.2%)	1,311 (38.3%)	
Moderately important	96 (14.2%)	389 (9.5%)	499 (14.6%)	
Slightly important	59 (8.7%)	114 (2.8%)	145 (4.2%)	
Not at all important	16 (2.4%)	23 (0.6%)	23 (0.7%)	
Rating of veterinarian knowledge				< 1.0e-4
Excellent	270 (40.0%)	2,149 (52.3%)	1,586 (46.3%)	
Good	244 (36.1%)	1,399 (34.1%)	1,329 (38.8%)	
Average	66 (9.8%)	275 (6.7%)	310 (9.0%)	
Poor	15 (2.2%)	21 (0.5%)	22 (0.6%)	
Very poor	7 (1.0%)	4 (0.1%)	3 (0.1%)	
NA	73 (10.8%)	259 (6.3%)	176 (5.1%)	
Rating of veterinarian bond with pet				<1.0e-4
Excellent	216 (32.0%)	1786 (43.5%)	1,210 (35.3%)	
Good	238 (35.3%)	1,597 (38.9%)	1,486 (43.4%)	
Average	122 (18.1%)	429 (10.4%)	521 (15.2%)	
Poor	20 (3.0%)	27 (0.7%)	24 (0.7%)	
Very poor	6 (0.9%)	9 (0.2%)	9 (0.3%)	
NA	73 (10.8%)	259 (6.3%)	176 (5.1%)	
Rating of veterinarian price				<1.0e-4
Excellent	158 (23.4%)	1,154 (28.1%)	843 (24.6%)	
Good	226 (33.5%)	1,614 (39.3%)	1,411 (41.2%)	
Average	175 (25.9%)	948 (23.1%)	872 (25.5%)	
Poor	27 (4.0%)	99 (2.4%)	100 (2.9%)	
Very poor	16 (2.4%)	33 (0.8%)	24 (0.7%)	
NA	73 (10.8%)	259 (6.3%)	176 (5.1%)	
Rating of veterinarian empathy toward pet				<1.0e-4
Excellent	238 (35.3%)	1971 (48.0%)	1,340 (39.1%)	
Good	252 (37.3%)	1,495 (36.4%)	1,474 (43.0%)	
Average	92 (13.6%)	344 (8.4%)	408 (11.9%)	
Poor	17 (2.5%)	28 (0.7%)	23 (0.7%)	
Very poor	3 (0.4%)	10 (0.2%)	5 (0.1%)	
NA	73 (10.8%)	259 (6.3%)	176 (5.1%)	
Rating of veterinarian explanations				<1.0e-4
Excellent	251 (37.2%)	2017 (49.1%)	1,433 (41.8%)	
Good	248 (36.7%)	1,521 (37.0%)	1,434 (41.9%)	
Average	78 (11.6%)	275 (6.7%)	344 (10.0%)	
Poor	18 (2.7%)	29 (0.7%)	37 (1.1%)	
Very poor	7 (1.0%)	6 (0.1%)	2 (0.1%)	
NA	73 (10.8%)	259 (6.3%)	176 (5.1%)	
Rating of veterinarian empathy toward owner				<1.0e-4
Excellent	208 (30.8%)	1791 (43.6%)	1,214 (35.4%)	
Good	258 (38.2%)	1,600 (39.0%)	1,529 (44.6%)	
Average	110 (16.3%)	411 (10.0%)	467 (13.6%)	
Poor	19 (2.8%)	32 (0.8%)	32 (0.9%)	
Very poor	7 (1.0%)	14 (0.3%)	8 (0.2%)	
NA	73 (10.8%)	259 (6.3%)	176 (5.1%)	
Rating of veterinarian incorporating owner decisions				<1.0e-4
Excellent	229 (33.9%)	1908 (46.5%)	1,311 (38.3%)	
Good	249 (36.9%)	1,545 (37.6%)	1,464 (42.7%)	
Average	97 (14.4%)	361 (8.8%)	440 (12.8%)	
Poor	19 (2.8%)	28 (0.7%)	26 (0.8%)	
Very poor	8 (1.2%)	6 (0.1%)	9 (0.3%)	
NA	73 (10.8%)	259 (6.3%)	176 (5.1%)	
Rating of veterinarian staff				<1.0e-4
Excellent	219 (32.4%)	1812 (44.1%)	1,318 (38.5%)	
Good	260 (38.5%)	1,598 (38.9%)	1,459 (42.6%)	
Average	103 (15.3%)	400 (9.7%)	431 (12.6%)	
Poor	15 (2.2%)	31 (0.8%)	37 (1.1%)	
Very poor	5 (0.7%)	7 (0.2%)	5 (0.1%)	
NA	73 (10.8%)	259 (6.3%)	176 (5.1%)	
Rating of veterinarian environment				< 1.0e-4
Excellent	209 (31.0%)	1720 (41.9%)	1,256 (36.7%)	
Good	259 (38.4%)	1,674 (40.8%)	1,497 (43.7%)	
Average	110 (16.3%)	417 (10.2%)	461 (13.5%)	
Poor	17 (2.5%)	30 (0.7%)	32 (0.9%)	
Very poor	7 (1.0%)	7 (0.2%)	4 (0.1%)	
NA	73 (10.8%)	259 (6.3%)	176 (5.1%)	

For brevity variables presented are a subset of all tested variables.

### 3.8 Budget for cat and cat product use

Significant differences were observed between the three clusters for the use of a range of care practices. Among the three clusters, CC3 had the most likely to use nutritional supplements for their cats (23.2% vs. 15.1% in CC1 and 19.8% in CC2). Regarding annual checkups, CC2 and CC3 were more likely to use this service (~52% in CC2 and CC3 vs. 41% in CC1). Similarly, screening services showed significant variation with CC1 (8.9%) being less likely to use screening while CC2 (14.3%) and CC3 (15.1%) were more likely to employ these services. Professional grooming services did differ significantly across clusters with cat owners in CC1 being less likely to use grooming services (27.1% vs. 35.1% in CC2 and 34.3% in CC3). Dentist visits varied significantly across clusters showing cluster differences, where CC1 (6.2%) and CC2 (9%) were less likely to use this service as compared to CC1 being more likely than expected to report using no services (15.6% vs. 10.3% in CC2 and 9.7% in CC3).

No significant difference among the three cat clusters (*p-*value threshold at 3.7e-4) was found for tooth brushing, monitoring device use, vaccination practices, skin medication use, or osteoarthritis medication use. Flea treatment was the only variable that could not be analyzed due to insufficient data for chi-squared testing, while medication for other ailments showed no significant cluster differences.

Significant differences were present across cat clusters for all variables with the exception of using a toothbrush, using monitoring devices, or using medications for osteoarthritis or skin-related conditions. Full differences can be seen in Supplementary File 4 Table 2.

### 3.9 Important veterinary qualities and perceived rating veterinary practitioner by cat owners

Significant differences were observed across the three cat-owner clusters for all aspects of veterinary care importance and ratings. CC1 was more likely than expected to rate the following as “not all important”: veterinary bond with cats (6.2% vs. 0.7% in CC2 and 1.4% in CC3) and empathy toward their cat (3.3% vs. 0.5% in CC2 and 0.7% in CC3). CC1 was less likely to rate the following qualities as “very important”: veterinarian explanations (50.7% vs. 63.8% in CC2 and 51.2% in CC3), clinic environment (39.1% in CC1, 53.0% in CC2, and 42.3% in CC3), owner decision-making respect (43.3% in CC1, 55.2% in CC2, and 44% in CC3), knowledge of veterinary staff (46.4% in CC1, 58.7% in CC2, and 49.9% in CC3) and empathy toward owners (37.3% in CC1, 51.8% in CC2, and 39.9% in CC3). CC2 tended to have the greatest proportion of members rate these qualities as “very important” while CC3 was in-between CC1 and CC2.

CC1 was more likely to rate veterinary pricing as “poor” (4.0% vs. 2.4% in CC2 and 2.9% in CC3) or “very poor” (2.4% vs. 0.8% in CC2 and 0.7% in CC3) than expected, but, along with participants in CC3, were less likely than expected to rate price as “very important” (CC1 36.7% and CC3 35.6% vs. 42.9% in CC2).

Significant differences were observed across clusters for multiple attitudes toward veterinary care. Notably, perceived veterinary knowledge varied significantly across clusters, with CC1 reporting more “average” categorisations (9.8%) and fewer “excellent” (40%) ratings than expected, while CC2 showed an abundance of “excellent” (52.3%) ratings. Significant differences were seen across groups in veterinarian visits per year. Though CC1 had the highest proportion of five visits a year or more (7.4, 6.0% in CC2, 6.9% in CC3), they also had the highest proportion of never taking their cat (11.6, 7.2% in CC2, 5.7% in CC3).

Significant differences were also seen across clusters regarding the importance and rating of veterinarian qualities. CC2 was significantly different across variables, with the exception of rating veterinarian price. Likewise, CC3 showed significantly higher average scores in these variables when compared to CC1.

### 3.10 Perceived impact on owner’s health

Significant differences were observed across clusters in respect of how cats were perceived to impact owners’ health and wellbeing. Most notably, clusters differed strongly in their responses to how cats help emotionally, including answering ‘yes’ to comfort when sad (35.6% in CC1, 58% in CC2, 42.6% in CC3), ‘yes’ to calming stress (39.1% in CC1, 55.3% in CC2, 43.6% in CC3), and ‘yes’ to generally making their owner happy (48% in CC1, 72.3% in CC2, 60.7% in CC3). In each case, CC2 reported these benefits more often than expected, while CC1 and CC3 were less likely to report them than expected.

Broader health-related impacts also showed strong variation. The impact of cat ownership on physical and mental health also differed by cluster. CC2 (21.9%) were more likely to report that both physical and mental health improved than expected. CC1 (49.3%) and CC2 (59.8%) were also more likely to report that *only* their mental health wellbeing improved than expected. CC1 (9.6%) and CC3 (12.8%) reported that *only* their physical health improved than expected. The belief that cats improve one’s own health differed significantly with CC2 (41.5%) stating their cat improved their health more than expected and higher than CC1 (27.0%) and CC3 (34.7%).

Cats were more likely to be seen as decreasing loneliness (53.6% in CC2 vs. 37.5% in CC1 and 43.2% in CC3) and providing a sense of purpose (47.4% in CC2 vs. 28.0% in CC1 and 29.8% in CC3), as well as adding happiness (65.9% in CC2 vs. 44.1% in CC1 and 56.5% in CC3) for members of CC2. Importantly, selecting “None of these benefits” option showed substantial divergence, with over-representation in CC1 (9.3%) compared to CC2 (1.5%) and CC3 (2.5%). Lastly, nonsignificant differences were observed in reported increases in physical activity, improving community connection, and better health as recognized by a doctor.

Significant differences in the average response were present across almost all health-related variables with the exception of improving activity levels and providing a greater sense of community. CC2 tended to have the highest average scores, with significant differences than CC1 and CC3 signifying a greater likelihood of reporting these benefits from owning their cat.

## 4 Discussion

Here we have studied the human-animal bond, by taking a data-driven approach to stratifying the pet-owner population, thus providing insight, in to not only factors that may influence a strong or weak bond, but how these bonds may be associated with different behaviors, care practices, product purchases and health perceptions. This study provides further evidence of the heterogeneity present among pet owners, not only within a specific pet ownership population (i.e., cats or dogs), but also between ownership of different pets. This offers the opportunity to understand the value of companion animals and their effect on human wellness more fully, taking into account gender and age, in a One Health paradigm.

Globally, there exists a distinction between individuals expressing stronger emotional connections with their pets compared to a more pragmatic (less emotional) consideration of the bond. Clusters of participants showing stronger emotional connections (C2 for dog owners, CC2 and CC3 for cat owners) were individuals that were more likely to conceptualize their pet as a child, reporting a higher willingness to spend money, utilize more services for their companion animal, and report greater mental and physical benefits. These clusters tended to have a higher proportion of “excellent” ratings for their veterinarians, C1 and CC1 displayed a more pragmatic nature, reporting higher likelihood for the pet to have a “working purpose” (e.g., guard/service dog or pest control), and reported a comparatively decreased willingness to spend money and less physical or mental benefits. Individuals with higher emotional connections may be more likely to anthropomophise their pet (e.g., viewing their pet as a child). While previous research (25) suggests anthropomoprhising can lead to higher bonds, this action may have negative effects on the pet such as use of clothing leading to restricted thermoregulation or dietary modifications, as well impacting the emotional wellbeing of owners (26). Future research will be needed to see how high HABSCORES can influence anthropomorphism of pets and the results of this practice.

Interestingly, no consistent pattern emerged between cluster membership and demographics across pet owners, with the exception of gender. Females were more likely to be represented by the cluster with the highest HABSCORE across both pet types (56.4% in C2 of dog owners, and 100% in CC3 of cat owners), echoing previous research collated by a narrative review finding that a small male/female difference in attachment to companion animals (27) and females showing greater empathy toward animals (25).

Country of origin and age demographics differed in regard to cluster membership for cat and dog owners. A further point of interest was the higher heterogeneity identified in cat owners (three clusters compared to two). Dog owners with higher HABSCOREs were more likely to be older (37.3% from ages 45 and up in C2 compared to 32.9% in C1), and more likely to be from “western” countries such as Germany, Spain, UK, and US. Cat owners in the lowest HABSCORE group (CC1) were more likely to be very young (18–24), while more likely to be older in CC3 compared to CC2 suggesting a potential age and gender interaction in cat owners. Cat owners also showed a different demographic grouping based on country. While some patterns remained consistent between pet owners (a larger proportion of Mexican residents in lower HABSCORE cluster, and a trend toward larger representation in western countries in higher HABSCORE cluster), cat owners did see differences in representation from China and Japan (higher proportion in higher HABSCORE compared to the inverse in dog owners). These findings are in contrast to previous research which has reported similar human-animal bonds across different ethnicities, both within a country (noting that white and American Indian participants were more likely to have companion animals (16)) and between countries (U. S. and Mexico) (17).

These country level differences highlight that the views expressed within each cluster are potentially influenced by a participant’s cultural norms. Previous research has highlighted that human-pet dynamics and bonds can vary across cultures (28, 29). Cultural norms can also contribute a mediating effect to other variables that impact anthropomorphism and attitudes toward wildlife, such as modernisation (30). Modernisation was found to have a positive effect on anthropomorphism in countries such as Spain, but a negative effect in Mexico. This may be contributing to the differences in the distribution of these two, and other countries across clusters in this paper. Past research conducted on the HABSCORE provides further clarity on this relationship as scores were unique across the different countries, with the United States scoring the highest (59.1) and Mexico the lowest (53.6). The findings in this current work may be the in-part result of such cultural contexts rather than psychological distinctions.

While a difference exists between the two cluster groupings, both populations exhibit caring attitudes toward their pets. This is exhibited in the non-significant differences in the responses to noticing issues or consulting veterinarians about a range of health conditions in their pet, ranging from weight gain, limping, difficulty going up and downstairs, and others among cat and dog owners, except for dog owners noticing separation anxiety and body odour as well as how participants assess the importance of and rate their veterinarians’ quality. Though significant differences are present, combining the “very important” and “important” categories broadly results in similar overall percentages, suggesting that most responses across all clusters are in these categories. This pattern is also demonstrated when combining the “money is no object” and “I spend a moderate amount on my pet” responses to budget. Though the proportion of responses were different between clusters, the pattern of responses did show some similarity. It is unclear whether this is indicative of a baseline level of responsibility or care that all pet owners feel toward their pets, or if this potentially due to bias in the sample as individuals responding to a survey of pet ownership may have higher bonds with their pets.

Previous research has suggested that the human-animal bond should not necessarily be characterized into a simple positive-caring and negative-abusive model (25). Rather it is potentially more complex and influenced by three human psychological mechanisms: empathy, attachment, and anthropomorphism. The findings of this current research do not support nor dispute this notion. While these findings provide evidence of the simplified positive-caring and negative-abusive model this may be impacted by the broad analytical approach and potential sampling bias as participants of this survey may have a higher affinity to animals on average. However, the HABSCORE does provide measures across these three constructs (i.e., empathy, attachment, and anthropomorphism), providing future avenues of research around these constructs and their impact not only on the human-animal bond, particularly in more general samples.

This study also provides further findings for the role of human-animal bond within a One Health paradigm. Previous research highlighted the impacts of pet ownership across four key areas including heart disease, cancer, autism, and economics (31). The authors noted not only positive health impacts, but also economic savings as the result of few doctor visits (31). The HABSCORE outcome is linked to positive health outcomes, providing initial scope for its role in a One Health paradigm. Additional research, particularly alongside standardized health outcomes, is required to understand how the HABSCORE can contribute to the integrated health of people, animals, and their shared environment.

This study is strengthened by a large, multi-national sample. However, it is not without limitations. As noted above, participants responding to this survey may be biased to have higher levels human-animal bonds and thus are not representative of the general pet-owner population. Secondly, findings related to mental and physical health are based on self-reported data rather than standardized health or medical assessments. Though previous research has shown that pet owners, specifically dog owners, are more likely to meet their physical activity guidelines, the literature around mental health has been less clear, with one study finding no impact on loneliness (32). While the health benefits of pet ownership in general are understood (33), the health benefits related to a strong human-animal bond are mixed particularly as it relates to mental health and attachment style (34). Future research could include standardized outcomes, such as the Patient Health Questionnaire for Depression and Anxiety (PHQ-4) (35) or Short Form Health Survey (36) for more robust evidence on the physical and mental health improvements of pet ownership and strong human-animal bonds, addressing this study’s limitations on self-reported outcome measures. Further, although variables were converted to a homogenous numeric form, the inclusion of a mixture of types of variables, e.g., gender and country, could impact the modeling approach. However, a sensitivity analysis using only HABSCORE questions and age (all ordinal variables) as input variables for the clustering model resulted in similar cluster characteristics and associations (see Supplementary Files 5, for dogs and Supplementary Files 6 for cats). Lastly, due to the observational nature of this survey we are unable to identify any causal impacts of pet ownership.

## 5 Conclusion

This study has taken a data-driven approach, based on a robust, multinational dataset. It provides further evidence of the heterogeneity between and within owners of certain pet types. Pet owners could be broadly split into two categories – those who with stronger emotional connections to their pet (more likely to report pet is like a child, higher benefits to physical and mental health, higher willingness to spend money), and more pragmatic pet owners. However, these differences do not seem to indicate any potential neglectful ownership qualities. Though this study has many strengths, future studies can improve by using standardized health outcome measures, pet health outcomes, and more specificity on budget (e.g., percent of income spent).

## Data Availability

The data analyzed in this study is subject to the following licenses/restrictions: The data presented in this article was made available by partnership with Human Animal Bond Research Institute and Zoetis and is not yet publicly available. Requests to access these datasets should be directed to n.geifman@surrey.ac.uk.
