# Supplementary material for: Companion animal owner “types” identified using a large-scale international assessment of the human-animal bond
Source: Front Vet Sci. 2026 May 12;13:1748135. doi: 10.3389/fvets.2026.1748135 (PMC13201171; doi:10.3389/fvets.2026.1748135)
Supplement: Supplementary file 2 [file Supplementary_file_2.pdf]

3 Regression

3.1 Multiple regression

Hide

```
m1 <- lm(habscore ~ S3 + AgeDum + FinalGroup + S1 + Q2 + Q3, data = d3)
summary(m1)
```

```
Call:
lm(formula = habscore ~ S3 + AgeDum + FinalGroup + S1 + Q2 +
    Q3, data = d3)

Residuals:
    Min      1Q  Median      3Q     Max
-48.110  -4.559   0.744   5.529  28.453

Coefficients:
                             Estimate Std. Error t value Pr(>|t|)
(Intercept)                  55.01751    0.32874 167.360  < 2e-16 ***
S3Male                       -1.12011    0.11525  -9.719  < 2e-16 ***
S3Other Gender Category      -0.20267    1.00867  -0.201 0.840753
S3Prefer Not to Say           0.75640    1.44674   0.523 0.601096
AgeDum25-34                  -0.06979    0.17930  -0.389 0.697113
AgeDum35-44                  -0.70044    0.18442  -3.798 0.000146 ***
AgeDum45-54                  -0.63364    0.19640  -3.226 0.001256 **
AgeDum55-64                  -0.64262    0.21945  -2.928 0.003412 **
AgeDum65+                    -1.38742    0.26592  -5.217 1.83e-07 ***
FinalGroupDog                 1.37437    0.11816  11.631  < 2e-16 ***
S1Brazil                      0.11063    0.30107   0.367 0.713275
S1China                      -0.15892    0.30291  -0.525 0.599837
S1France                     -0.31627    0.29874  -1.059 0.289766
S1Germany                     0.14713    0.30090   0.489 0.624879
S1Japan                      -2.04211    0.30727  -6.646 3.09e-11 ***
S1Mexico                     -4.31553    0.30088 -14.343  < 2e-16 ***
S1Spain                       0.24166    0.30028   0.805 0.420956
S1UK                          0.08875    0.29741   0.298 0.765384
S1USA                         0.90585    0.29731   3.047 0.002316 **
Q2A friend                    2.00800    0.25957   7.736 1.08e-14 ***
Q2Family member               3.24322    0.19390  16.726  < 2e-16 ***
Q2Just a pet                 -9.79304    0.36968 -26.491  < 2e-16 ***
Q2Like a child                5.62891    0.19745  28.508  < 2e-16 ***
Q3Emotional Support Animal    1.11099    0.18040   6.158 7.50e-10 ***
Q3Guard dog                  -3.49153    0.38546  -9.058  < 2e-16 ***
Q3Keep Mice Out/Pest Control -4.54152    0.52285  -8.686  < 2e-16 ***
Q3Other (please specify)     -0.61463    0.30097  -2.042 0.041148 *
Q3Service Animal             -0.67341    0.59373  -1.134 0.256722
---
Signif. codes:  0 ‘***’ 0.001 ‘**’ 0.01 ‘*’ 0.05 ‘.’ 0.1 ‘ ’ 1

Residual standard error: 7.772 on 19159 degrees of freedom
Multiple R-squared:  0.1894,    Adjusted R-squared:  0.1883
F-statistic: 165.8 on 27 and 19159 DF,  p-value: < 2.2e-16
```

Hide

```
anova(m1)
```

```
Analysis of Variance Table

Response: habscore
               Df  Sum Sq Mean Sq F value    Pr(>F)
S3              3   19344    6448 106.745 < 2.2e-16 ***
AgeDum          5   11870    2374  39.301 < 2.2e-16 ***
FinalGroup      1    9074    9074 150.216 < 2.2e-16 ***
S1              9   51140    5682  94.069 < 2.2e-16 ***
Q2              4  166097   41524 687.429 < 2.2e-16 ***
Q3              5   12947    2589  42.866 < 2.2e-16 ***
Residuals   19159 1157306      60
---
Signif. codes:  0 ‘***’ 0.001 ‘**’ 0.01 ‘*’ 0.05 ‘.’ 0.1 ‘ ’ 1
```

Hide

```
check_model(m1)
```

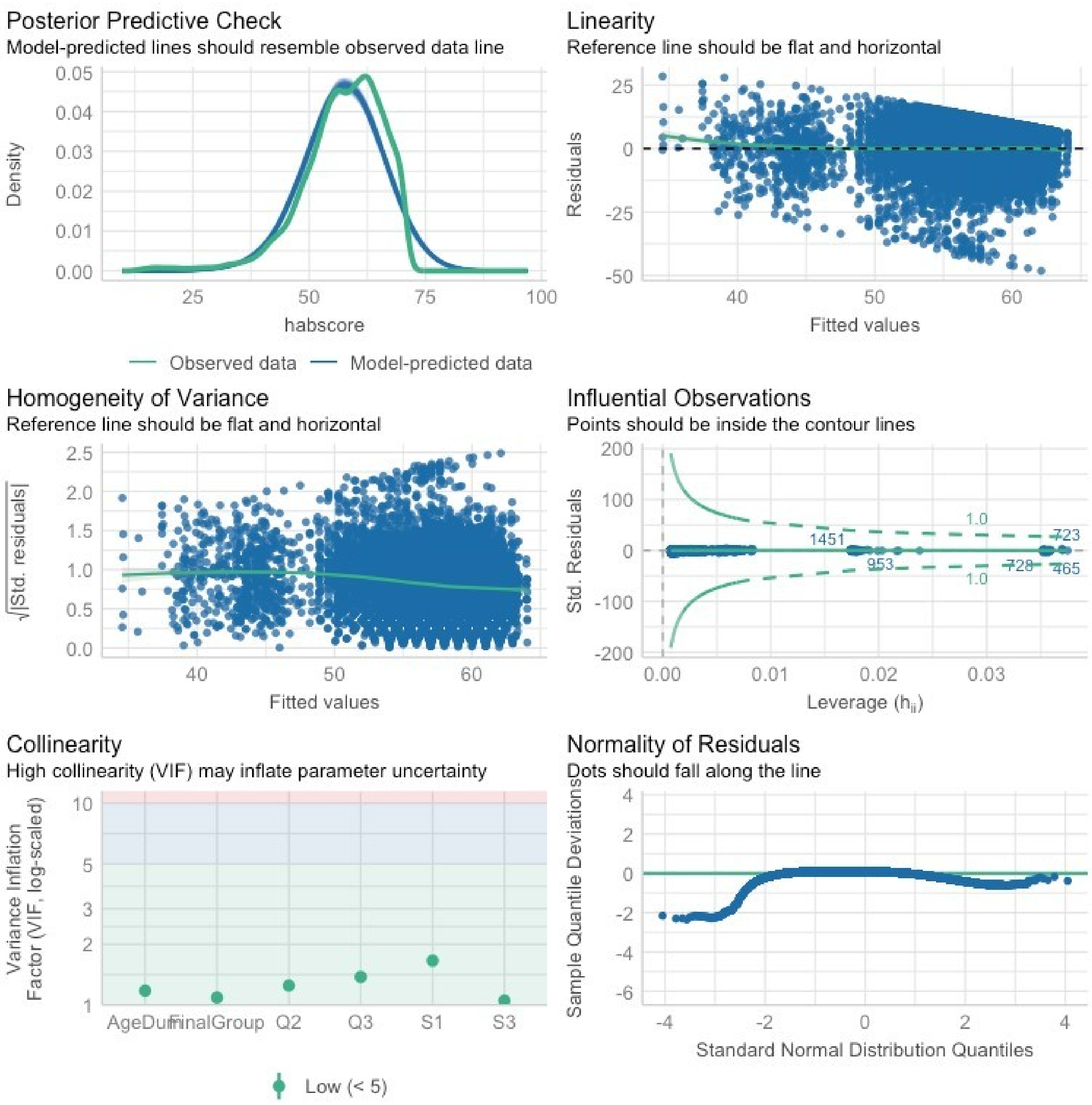

Hide

```
NA
NA
```

3.2 Interaction between Gender and Pet Purpose

Hide

```
m2 <- lm(habscore ~ S3 + AgeDum + FinalGroup + S1 + Q2 + Q3 + S3*Q3, data = d3)
summary(m2)
```

```
Call:
lm(formula = habscore ~ S3 + AgeDum + FinalGroup + S1 + Q2 +
    Q3 + S3 * Q3, data = d3)

Residuals:
    Min      1Q  Median      3Q     Max
-48.117  -4.548   0.735   5.516  27.565

Coefficients: (2 not defined because of singularities)
                                         Estimate Std. Error t value Pr(>|t|)
(Intercept)                              55.03250    0.33001 166.761  < 2e-16 **
*
S3Male                                   -1.15561    0.12896  -8.961  < 2e-16 **
*
S3Other Gender Category                   0.82494    1.19068   0.693 0.488423
S3Prefer Not to Say                       0.21014    1.69918   0.124 0.901578
AgeDum25-34                              -0.06910    0.17939  -0.385 0.700095
AgeDum35-44                              -0.69294    0.18456  -3.755 0.000174 **
*
AgeDum45-54                              -0.63259    0.19647  -3.220 0.001285 **
AgeDum55-64                              -0.62955    0.21955  -2.867 0.004143 **
AgeDum65+                                -1.38127    0.26605  -5.192 2.10e-07 **
*
FinalGroupDog                             1.37985    0.11821  11.673  < 2e-16 **
*
S1Brazil                                  0.10908    0.30126   0.362 0.717300
S1China                                  -0.17514    0.30328  -0.577 0.563621
S1France                                 -0.32034    0.29888  -1.072 0.283810
S1Germany                                 0.15422    0.30122   0.512 0.608659
S1Japan                                  -2.07289    0.30778  -6.735 1.69e-11 **
*
S1Mexico                                 -4.32418    0.30099 -14.367  < 2e-16 **
*
S1Spain                                   0.23643    0.30042   0.787 0.431287
S1UK                                      0.07889    0.29755   0.265 0.790904
S1USA                                     0.89976    0.29750   3.024 0.002495 **
Q2A friend                                2.01735    0.25975   7.767 8.47e-15 **
*
Q2Family member                           3.24371    0.19398  16.722  < 2e-16 **
*
Q2Just a pet                             -9.82249    0.37025 -26.530  < 2e-16 **
*
Q2Like a child                            5.62580    0.19754  28.479  < 2e-16 **
*
Q3Emotional Support Animal                0.99824    0.24083   4.145 3.41e-05 **
*
Q3Guard dog                              -3.06150    0.60162  -5.089 3.64e-07 **
*
Q3Keep Mice Out/Pest Control             -6.59349    0.95179  -6.927 4.42e-12 **
*
Q3Other (please specify)                 -0.52520    0.39027  -1.346 0.178401
Q3Service Animal                         -0.28925    0.84153  -0.344 0.731059
```

```
S3Male:Q3Emotional Support Animal                    0.26962    0.34636   0.778 0.436320
S3Other Gender Category:Q3Emotional Support Animal   -3.49160    3.00328  -1.163 0.245009
S3Prefer Not to Say:Q3Emotional Support Animal        0.55867    5.75857   0.097 0.922715
S3Male:Q3Guard dog                                   -0.67983    0.77100  -0.882 0.377925
S3Other Gender Category:Q3Guard dog                  -8.33001    7.88791  -1.056 0.290959
S3Prefer Not to Say:Q3Guard dog                       0.80246    5.78354   0.139 0.889650
S3Male:Q3Keep Mice Out/Pest Control                   2.99091    1.12871   2.650 0.008059 **
S3Other Gender Category:Q3Keep Mice Out/Pest Control -5.01061    5.70444  -0.878 0.379754
S3Prefer Not to Say:Q3Keep Mice Out/Pest Control           NA         NA      NA       NA
S3Male:Q3Other (please specify)                      -0.20635    0.57677  -0.358 0.720528
S3Other Gender Category:Q3Other (please specify)     -1.31745    3.69332  -0.357 0.721311
S3Prefer Not to Say:Q3Other (please specify)          2.98120    4.25798   0.700 0.483846
S3Male:Q3Service Animal                              -0.70726    1.18789  -0.595 0.551589
S3Other Gender Category:Q3Service Animal             -5.55917    7.91281  -0.703 0.482343
S3Prefer Not to Say:Q3Service Animal                       NA         NA      NA       NA
---
Signif. codes:  0 '***' 0.001 '**' 0.01 '*' 0.05 '.' 0.1 ' ' 1

Residual standard error: 7.772 on 19146 degrees of freedom
Multiple R-squared:   0.19, Adjusted R-squared:  0.1883
F-statistic: 112.3 on 40 and 19146 DF,  p-value: < 2.2e-16
```

Hide

```
anova(m2)
```

```
Analysis of Variance Table

Response: habscore
             Df  Sum Sq Mean Sq  F value Pr(>F)
S3            3   19344    6448 106.7472 <2e-16 ***
AgeDum        5   11870    2374  39.3019 <2e-16 ***
FinalGroup    1    9074    9074 150.2197 <2e-16 ***
S1            9   51140    5682  94.0711 <2e-16 ***
Q2            4  166097   41524 687.4460 <2e-16 ***
Q3            5   12947    2589  42.8669 <2e-16 ***
S3:Q3        13     814      63   1.0368 0.4116
Residuals 19146 1156491      60
---
Signif. codes:  0 '***' 0.001 '**' 0.01 '*' 0.05 '.' 0.1 ' ' 1
```

Hide

```
check_model(m2)
```

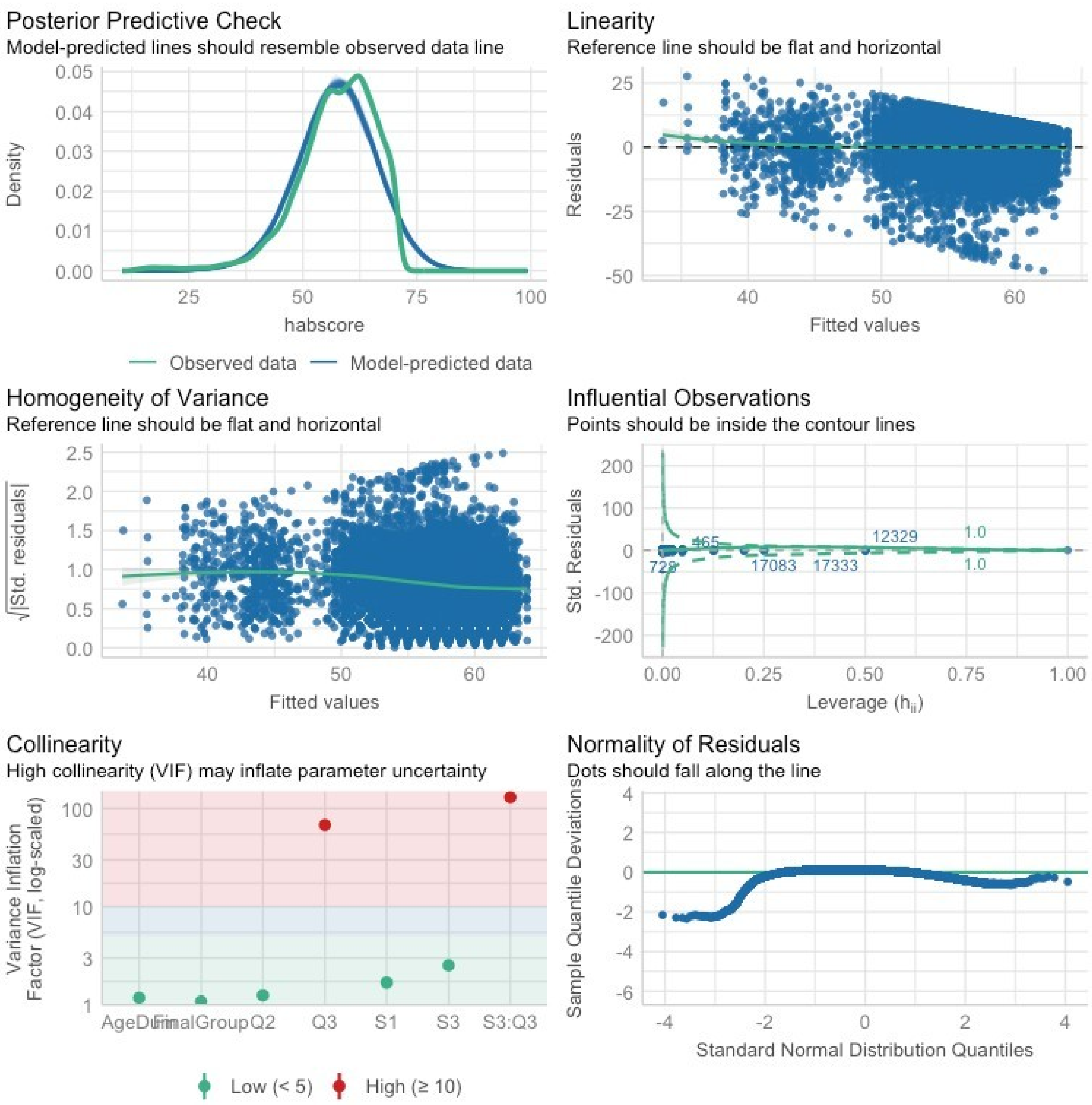

3.3 Interaction between Gender and Country

Hide

```
m3 <- lm(habscore ~ S3 + AgeDum + FinalGroup + S1 + Q2 + Q3 +S3*S1, data = d3)
summary(m3)
```

```
Call:
lm(formula = habscore ~ S3 + AgeDum + FinalGroup + S1 + Q2 +
    Q3 + S3 * S1, data = d3)

Residuals:
    Min      1Q  Median      3Q     Max
-48.315  -4.514   0.702   5.518  40.518

Coefficients: (1 not defined because of singularities)
                                 Estimate Std. Error t value Pr(>|t|)
(Intercept)                      55.07981    0.39625 139.003  < 2e-16 ***
S3Male                           -1.24082    0.48310  -2.568 0.010223 *
S3Other Gender Category          -1.83176    4.50070  -0.407 0.684018
S3Prefer Not to Say              -9.70581    7.77414  -1.248 0.211873
AgeDum25-34                      -0.05882    0.17974  -0.327 0.743471
AgeDum35-44                      -0.67981    0.18481  -3.678 0.000235 ***
AgeDum45-54                      -0.63110    0.19660  -3.210 0.001329 **
AgeDum55-64                      -0.60246    0.22051  -2.732 0.006299 **
AgeDum65+                        -1.34393    0.26753  -5.023 5.12e-07 ***
FinalGroupDog                     1.40082    0.11835  11.837  < 2e-16 ***
S1Brazil                          0.25562    0.41957   0.609 0.542366
S1China                          -0.44910    0.40808  -1.101 0.271112
S1France                         -0.22257    0.41073  -0.542 0.587896
S1Germany                         0.20949    0.40889   0.512 0.608419
S1Japan                          -3.03107    0.43012  -7.047 1.89e-12 ***
S1Mexico                         -4.18291    0.41299 -10.128  < 2e-16 ***
S1Spain                           0.49436    0.41475   1.192 0.233296
S1UK                              0.23631    0.41548   0.569 0.569522
S1USA                             0.64874    0.40891   1.587 0.112640
Q2A friend                        1.98147    0.25956   7.634 2.38e-14 ***
Q2Family member                   3.22518    0.19393  16.630  < 2e-16 ***
Q2Just a pet                     -9.77942    0.36966 -26.455  < 2e-16 ***
Q2Like a child                    5.59818    0.19758  28.334  < 2e-16 ***
Q3Emotional Support Animal        1.05452    0.18095   5.828 5.71e-09 ***
Q3Guard dog                      -3.51697    0.38576  -9.117  < 2e-16 ***
Q3Keep Mice Out/Pest Control     -4.57353    0.52277  -8.749  < 2e-16 ***
Q3Other (please specify)         -0.52036    0.30167  -1.725 0.084561 .
Q3Service Animal                 -0.67924    0.59432  -1.143 0.253099
S3Male:S1Brazil                  -0.28495    0.59484  -0.479 0.631919
S3Other Gender Category:S1Brazil  -4.67202    7.10490  -0.658 0.510818
S3Prefer Not to Say:S1Brazil      10.46138    9.52536   1.098 0.272103
S3Male:S1China                    0.70453    0.59979   1.175 0.240160
S3Other Gender Category:S1China    4.60287    8.98017   0.513 0.608266
S3Prefer Not to Say:S1China             NA         NA      NA       NA
S3Male:S1France                  -0.23612    0.59641  -0.396 0.692177
S3Other Gender Category:S1France  -3.96228    7.10569  -0.558 0.577110
S3Prefer Not to Say:S1France      15.23099    8.69350   1.752 0.079790 .
S3Male:S1Germany                 -0.15299    0.59415  -0.258 0.796795
S3Other Gender Category:S1Germany  2.10852    5.94955   0.354 0.723043
S3Prefer Not to Say:S1Germany      7.84933    9.52087   0.824 0.409703
S3Male:S1Japan                    1.77857    0.59645   2.982 0.002868 **
S3Other Gender Category:S1Japan    6.12115    5.13408   1.192 0.233176
```

```
S3Prefer Not to Say:S1Japan       15.31184    8.24882   1.856 0.063434 .
S3Male:S1Mexico                   -0.27432    0.59606  -0.460 0.645369
S3Other Gender Category:S1Mexico  -0.24538    5.19735  -0.047 0.962344
S3Prefer Not to Say:S1Mexico      -4.92952    8.97793  -0.549 0.582963
S3Male:S1Spain                    -0.56864    0.59497  -0.956 0.339209
S3Other Gender Category:S1Spain    0.62790    5.07900   0.124 0.901612
S3Prefer Not to Say:S1Spain       11.03863    8.51727   1.296 0.194981
S3Male:S1UK                       -0.34076    0.59860  -0.569 0.569188
S3Other Gender Category:S1UK       1.37350    5.37916   0.255 0.798465
S3Prefer Not to Say:S1UK          14.82251   10.99089   1.349 0.177476
S3Male:S1USA                       0.53868    0.59737   0.902 0.367201
S3Other Gender Category:S1USA      2.71148    5.07978   0.534 0.593501
S3Prefer Not to Say:S1USA         10.73857    8.97807   1.196 0.231677
---
Signif. codes:  0 ‘***’ 0.001 ‘**’ 0.01 ‘*’ 0.05 ‘.’ 0.1 ‘ ’ 1

Residual standard error: 7.765 on 19133 degrees of freedom
Multiple R-squared:  0.1919,    Adjusted R-squared:  0.1897
F-statistic: 85.75 on 53 and 19133 DF,  p-value: < 2.2e-16
```

Hide

```
anova(m3)
```

```
Analysis of Variance Table

Response: habscore
             Df  Sum Sq Mean Sq  F value    Pr(>F)
S3            3   19344    6448 106.9306 < 2.2e-16 ***
AgeDum        5   11870    2374  39.3694 < 2.2e-16 ***
FinalGroup    1    9074    9074 150.4778 < 2.2e-16 ***
S1            9   51140    5682  94.2328 < 2.2e-16 ***
Q2            4  166097   41524 688.6275 < 2.2e-16 ***
Q3            5   12947    2589  42.9405 < 2.2e-16 ***
S3:S1        26    3582     138   2.2848 0.0002049 ***
Residuals 19133 1153723      60
---
Signif. codes:  0 ‘***’ 0.001 ‘**’ 0.01 ‘*’ 0.05 ‘.’ 0.1 ‘ ’ 1
```

Hide

```
check_model(m3)
```

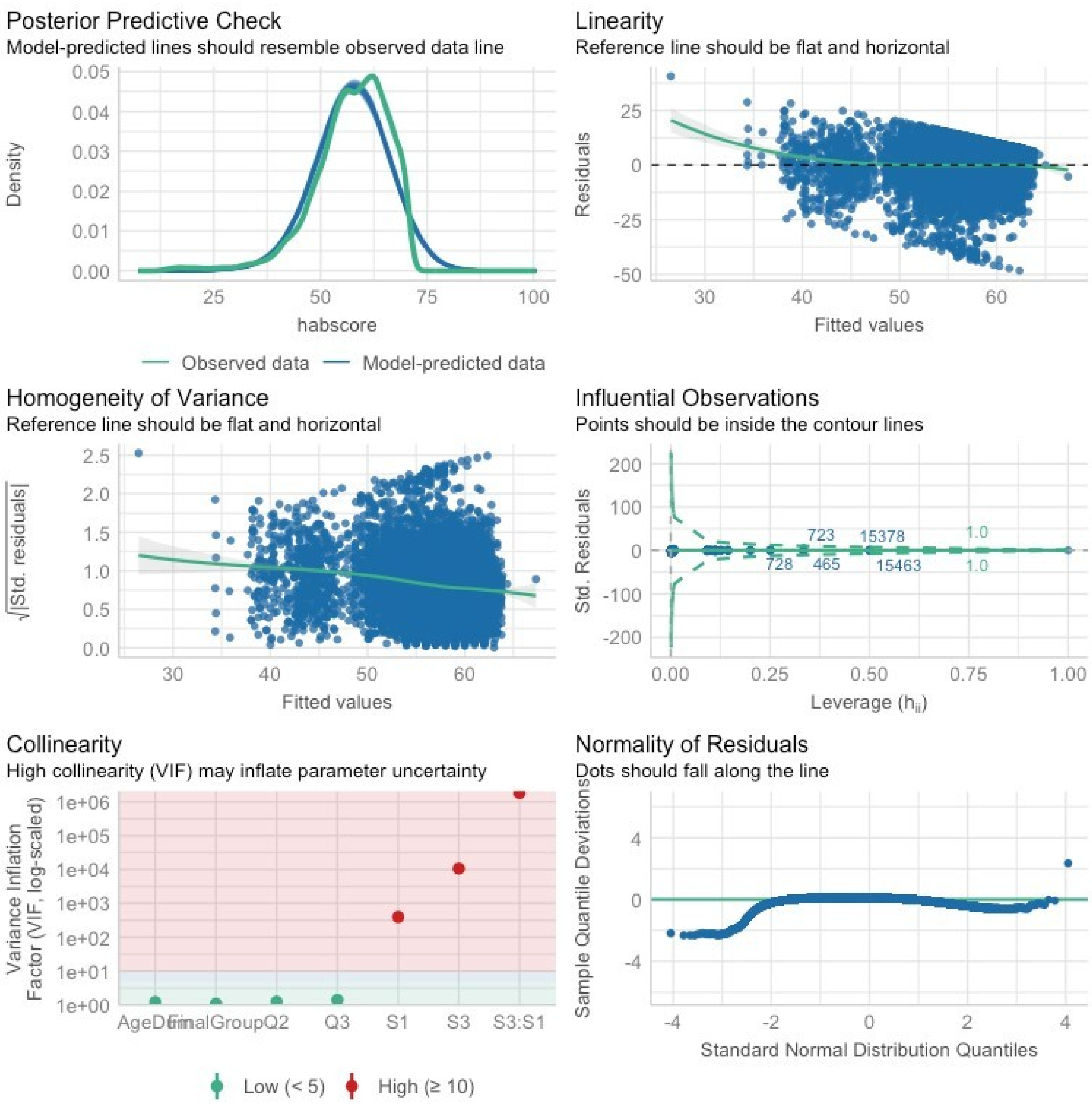

3.4 Full model

Hide

```
d3 %>%
  select(habscore, S3, AgeDum, FinalGroup, S1, Q2, Q3, Q5, Q7:Q11, Q14:Q20_9) -> d4

d5 <- d4 %>%
  mutate(across(Q19_1:Q20_9, ~ case_when(
                          . == "Strongly Disagree" ~ 1,
                          . == "Disagree" ~ 2,
                          . == "Neutral" ~ 3,
                          . == "Agree" ~ 4,
                          . == "Strongly Agree" ~ 5,
                          . == "Not at all important" ~ 1,
                          . == "Slightly important" ~ 2,
                          . == "Moderately important" ~ 3,
                          . == "Important" ~ 4,
                          . == "Very important" ~ 5,
                          . == "Very poor" ~ 1,
                          . == "Poor" ~ 2,
                          . == "Average" ~ 3,
                          . == "Good" ~ 4,
                          . == "Excellent" ~ 5)))

d5 = d5 %>% filter(complete.cases(.))

corr <- round(cor(d5[, c(1,19:36)]), 2)
ggcorrplot(corr,
           hc.order = TRUE, # re-order
           type = "lower", # lower triangle
           method = "circle", # use circle
   lab = TRUE)
```

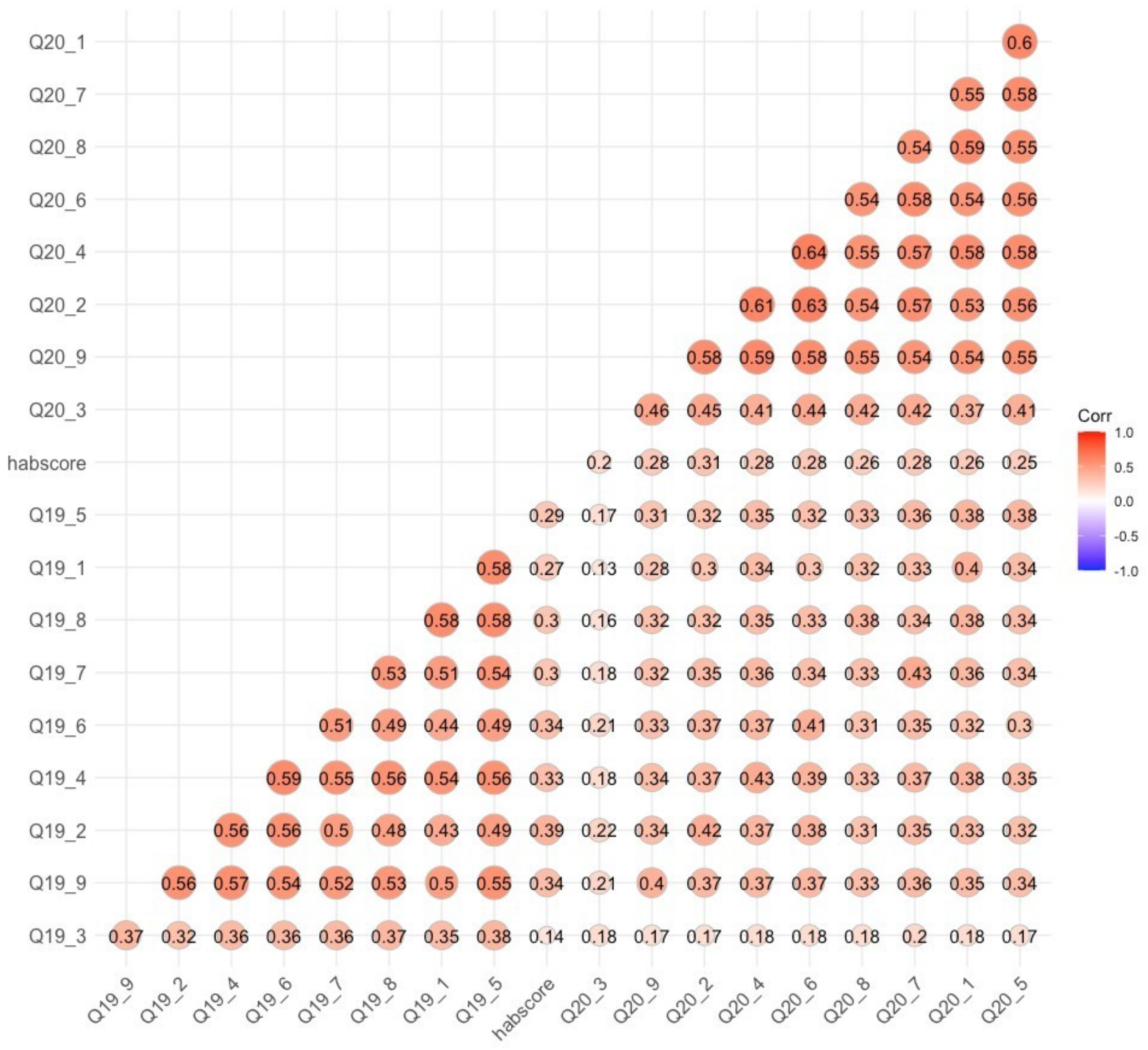

Hide

```
m4 <- lm(habscore ~ ., data = d5)
summary(m4)
```

```
Call:
lm(formula = habscore ~ ., data = d5)

Residuals:
    Min      1Q  Median      3Q     Max 
-54.788  -3.406   0.587   4.116  26.615 

Coefficients:
                                                     Estimate Std. Error t
value
(Intercept)                                          30.38301    0.64476
47.123
S3Male                                               -0.81070    0.10145
-7.991
S3Other Gender Category                              -0.09231    0.86918
-0.106
S3Prefer Not to Say                                   1.29096    1.27149
1.015
AgeDum25-34                                          -0.06362    0.15604
-0.408
AgeDum35-44                                          -0.14013    0.16250
-0.862
AgeDum45-54                                           0.15504    0.17487
0.887
AgeDum55-64                                           0.12469    0.19703
0.633
AgeDum65+                                            -0.42727    0.23971
-1.782
FinalGroupDog                                         0.92828    0.10844
8.560
S1Brazil                                             -1.47553    0.27688
-5.329
S1China                                              -0.87049    0.28688
-3.034
S1France                                             -0.48356    0.26421
-1.830
S1Germany                                            -0.86069    0.26758
-3.217
S1Japan                                               0.54930    0.28184
1.949
S1Mexico                                             -6.50901    0.27821
-23.396
S1Spain                                              -1.54402    0.26919
-5.736
S1UK                                                 -0.29830    0.26076
-1.144
S1USA                                                 0.35155    0.26151
1.344
Q2A friend                                            1.15163    0.22465
5.126
Q2Family member                                       1.93553    0.16861
11.480
```

Q2Just a pet -6.39494 0.33157
-19.287
Q2Like a child 3.52658 0.17313
20.370
Q3Emotional Support Animal 0.76229 0.15613
4.882
Q3Guard dog -1.85567 0.33684
-5.509
Q3Keep Mice Out/Pest Control -2.47510 0.47793
-5.179
Q3Other (please specify) 0.15266 0.26308
0.580
Q3Service Animal 0.11638 0.51140
0.228
Q5Friend / family member -0.49282 0.17516
-2.814
Q5He /she was a stray animal I found myself 0.18348 0.21605
0.849
Q5Other (please specify) -0.17278 0.36746
-0.470
Q5Pet store -0.23170 0.20447
-1.133
Q5Private individual other than friends & family -0.41140 0.18570
-2.215
Q5Puppy/kitten from own dog/cat 0.24296 0.32515
0.747
Q5Shelter / rescue organization -0.11149 0.17709
-0.630
Q7Yes, I did a lot of research 1.54487 0.15250
10.130
Q7Yes, I did some research 0.41810 0.12894
3.243
Q8I thought about the decision but not in detail -0.59028 0.12124
-4.869
Q8It was an impulse decision -0.05910 0.14652
-0.403
Q9I spend a moderate amount on my pet 0.72277 0.15132
4.776
Q9Money is no object when it comes to my pet 3.33743 0.16069
20.770
Q11#PETTAG1# is generally healthy 0.05191 0.16475
0.315
Q14I used to have pet health insurance for #PETTAG1# but don't anymore 0.12656 0.18634
0.679
Q14Yes, I currently have pet health insurance for #PETTAG1# 0.62187 0.12846
4.841
Q15_1Four times a year 0.19657 0.23115
0.850
Q15_1I have never taken him/her to see a veterinarian -0.26054 0.73419
-0.355
Q15_1Less than once a year -0.92522 0.30398
-3.044
Q15_1Once a year -0.39386 0.25304
-1.556

Q15_1Three times a year	0.19239	0.22823	0.843
Q15_1Twice a year	0.26401	0.23140	1.141
Q15_2Four times a year	0.20462	0.28847	0.709
Q15_2I have never taken him/her to see a veterinarian	-1.15596	0.34283	-3.372
Q15_2Less than once a year	-0.47666	0.30179	-1.579
Q15_2Once a year	-0.40756	0.27741	-1.469
Q15_2Three times a year	-0.09860	0.26967	-0.366
Q15_2Twice a year	-0.21042	0.26469	-0.795
Q16Yes	0.04181	0.14358	0.291
Q17Unsure	0.44176	0.12331	3.582
Q17Yes	0.96961	0.15119	6.413
Q18Unsure	0.83359	0.22374	3.726
Q18Yes, a lot more	1.93054	0.15774	12.238
Q18Yes, somewhat	1.25551	0.12869	9.756
Q19_1	0.31467	0.08590	3.663
Q19_2	1.24453	0.07440	16.728
Q19_3	-0.12503	0.05982	-2.090
Q19_4	0.54175	0.08585	6.310
Q19_5	0.03552	0.08850	0.401
Q19_6	0.36209	0.07357	4.922
Q19_7	0.35070	0.07782	4.507
Q19_8	0.40726	0.08475	4.805
Q19_9	0.44536	0.08168	5.452
Q20_1	0.19206	0.10366	1.853
Q20_2	0.57171	0.09836	5.813
Q20_3	0.25569	0.06943	3.683
Q20_4	0.22928	0.10373	2.210

```
Q20_5                                          0.06045    0.10223
0.591
Q20_6                                         -0.07327    0.10126
-0.724
Q20_7                                          0.31254    0.09723
3.214
Q20_8                                          0.07499    0.09502
0.789
Q20_9                                          0.17752    0.09655
1.839
                                                        Pr(>|t|)
(Intercept)                                            < 2e-16 ***
S3Male                                                1.41e-15 ***
S3Other Gender Category                               0.915425
S3Prefer Not to Say                                   0.309970
AgeDum25-34                                           0.683496
AgeDum35-44                                           0.388505
AgeDum45-54                                           0.375292
AgeDum55-64                                           0.526838
AgeDum65+                                             0.074689 .
FinalGroupDog                                          < 2e-16 ***
S1Brazil                                              9.99e-08 ***
S1China                                               0.002414 **
S1France                                              0.067233 .
S1Germany                                             0.001300 **
S1Japan                                               0.051315 .
S1Mexico                                               < 2e-16 ***
S1Spain                                               9.86e-09 ***
S1UK                                                  0.252657
S1USA                                                 0.178866
Q2A friend                                            2.99e-07 ***
Q2Family member                                        < 2e-16 ***
Q2Just a pet                                           < 2e-16 ***
Q2Like a child                                         < 2e-16 ***
Q3Emotional Support Animal                            1.06e-06 ***
Q3Guard dog                                           3.66e-08 ***
Q3Keep Mice Out/Pest Control                          2.26e-07 ***
Q3Other (please specify)                              0.561733
Q3Service Animal                                      0.819982
Q5Friend / family member                              0.004904 **
Q5He /she was a stray animal I found myself           0.395763
Q5Other (please specify)                              0.638209
Q5Pet store                                           0.257165
Q5Private individual other than friends & family      0.026748 *
Q5Puppy/kitten from own dog/cat                       0.454930
Q5Shelter / rescue organization                       0.528992
Q7Yes, I did a lot of research                         < 2e-16 ***
Q7Yes, I did some research                            0.001187 **
Q8I thought about the decision but not in detail      1.13e-06 ***
Q8It was an impulse decision                          0.686684
Q9I spend a moderate amount on my pet                 1.80e-06 ***
Q9Money is no object when it comes to my pet           < 2e-16 ***
Q11#PETTAG1# is generally healthy                     0.752688
Q14I used to have pet health insurance for #PETTAG1# but don't anymore 0.497019
```

```
Q14Yes, I currently have pet health insurance for #PETTAG1#            1.30e-06 ***
Q15_1Four times a year                                     0.395123
Q15_1I have never taken him/her to see a veterinarian               0.722696
Q15_1Less than once a year                                    0.002340 **
Q15_1Once a year                                         0.119607
Q15_1Three times a year                                     0.399262
Q15_1Twice a year                                        0.253922
Q15_2Four times a year                                     0.478140
Q15_2I have never taken him/her to see a veterinarian               0.000748 ***
Q15_2Less than once a year                                    0.114254
Q15_2Once a year                                         0.141803
Q15_2Three times a year                                     0.714634
Q15_2Twice a year                                        0.426639
Q16Yes                                              0.770890
Q17Unsure                                             0.000341 ***
Q17Yes                                              1.46e-10 ***
Q18Unsure                                             0.000195 ***
Q18Yes, a lot more                                        < 2e-16 ***
Q18Yes, somewhat                                          < 2e-16 ***
Q19_1                                              0.000250 ***
Q19_2                                               < 2e-16 ***
Q19_3                                              0.036611 *
Q19_4                                              2.85e-10 ***
Q19_5                                              0.688120
Q19_6                                              8.66e-07 ***
Q19_7                                              6.63e-06 ***
Q19_8                                              1.56e-06 ***
Q19_9                                              5.03e-08 ***
Q20_1                                              0.063930 .
Q20_2                                              6.25e-09 ***
Q20_3                                              0.000231 ***
Q20_4                                              0.027100 *
Q20_5                                              0.554333
Q20_6                                              0.469307
Q20_7                                              0.001310 **
Q20_8                                              0.430018
Q20_9                                              0.065993 .
---
Signif. codes:  0 ‘***’ 0.001 ‘**’ 0.01 ‘*’ 0.05 ‘.’ 0.1 ‘ ’ 1

Residual standard error: 6.57 on 18366 degrees of freedom
Multiple R-squared:  0.4057,	Adjusted R-squared:  0.4031 
F-statistic: 158.7 on 79 and 18366 DF,  p-value: < 2.2e-16
```

Hide

```
anova(m4)
```

```
Analysis of Variance Table

Response: habscore
             Df Sum Sq Mean Sq   F value    Pr(>F)
S3            3  18917    6306  146.1035 < 2.2e-16 ***
AgeDum        5   9888    1978   45.8200 < 2.2e-16 ***
FinalGroup    1   6649    6649  154.0681 < 2.2e-16 ***
S1            9  50560    5618  130.1655 < 2.2e-16 ***
Q2            4 141655   35414  820.5479 < 2.2e-16 ***
Q3            5   9951    1990   46.1139 < 2.2e-16 ***
Q5            7   5204     743   17.2254 < 2.2e-16 ***
Q7            2  35200   17600  407.7979 < 2.2e-16 ***
Q8            2  10418    5209  120.6950 < 2.2e-16 ***
Q9            2  70367   35183  815.2133 < 2.2e-16 ***
Q11           1     25      25    0.5766 0.4476574
Q14           2   6502    3251   75.3253 < 2.2e-16 ***
Q15_1         6  10182    1697   39.3208 < 2.2e-16 ***
Q15_2         6   1048     175    4.0466 0.0004666 ***
Q16           1    338     338    7.8239 0.0051613 **
Q17           2   8045    4022   93.1974 < 2.2e-16 ***
Q18           3  13340    4447  103.0286 < 2.2e-16 ***
Q19_1         1  52341   52341 1212.7662 < 2.2e-16 ***
Q19_2         1  55936   55936 1296.0636 < 2.2e-16 ***
Q19_3         1    572     572   13.2599 0.0002719 ***
Q19_4         1   9551    9551  221.2952 < 2.2e-16 ***
Q19_5         1   1568    1568   36.3214 1.705e-09 ***
Q19_6         1   3205    3205   74.2602 < 2.2e-16 ***
Q19_7         1   2665    2665   61.7453 4.123e-15 ***
Q19_8         1   1809    1809   41.9192 9.753e-11 ***
Q19_9         1   2177    2177   50.4381 1.275e-12 ***
Q20_1         1   5088    5088  117.8841 < 2.2e-16 ***
Q20_2         1   5263    5263  121.9373 < 2.2e-16 ***
Q20_3         1   1259    1259   29.1632 6.736e-08 ***
Q20_4         1    521     521   12.0792 0.0005110 ***
Q20_5         1    110     110    2.5385 0.1111192
Q20_6         1      0       0    0.0032 0.9551529
Q20_7         1    536     536   12.4136 0.0004272 ***
Q20_8         1     45      45    1.0463 0.3063722
Q20_9         1    146     146    3.3804 0.0659928 .
Residuals 18366 792649      43
---
Signif. codes:  0 ‘***’ 0.001 ‘**’ 0.01 ‘*’ 0.05 ‘.’ 0.1 ‘ ’ 1
```

Hide

```
check_model(m4)
```

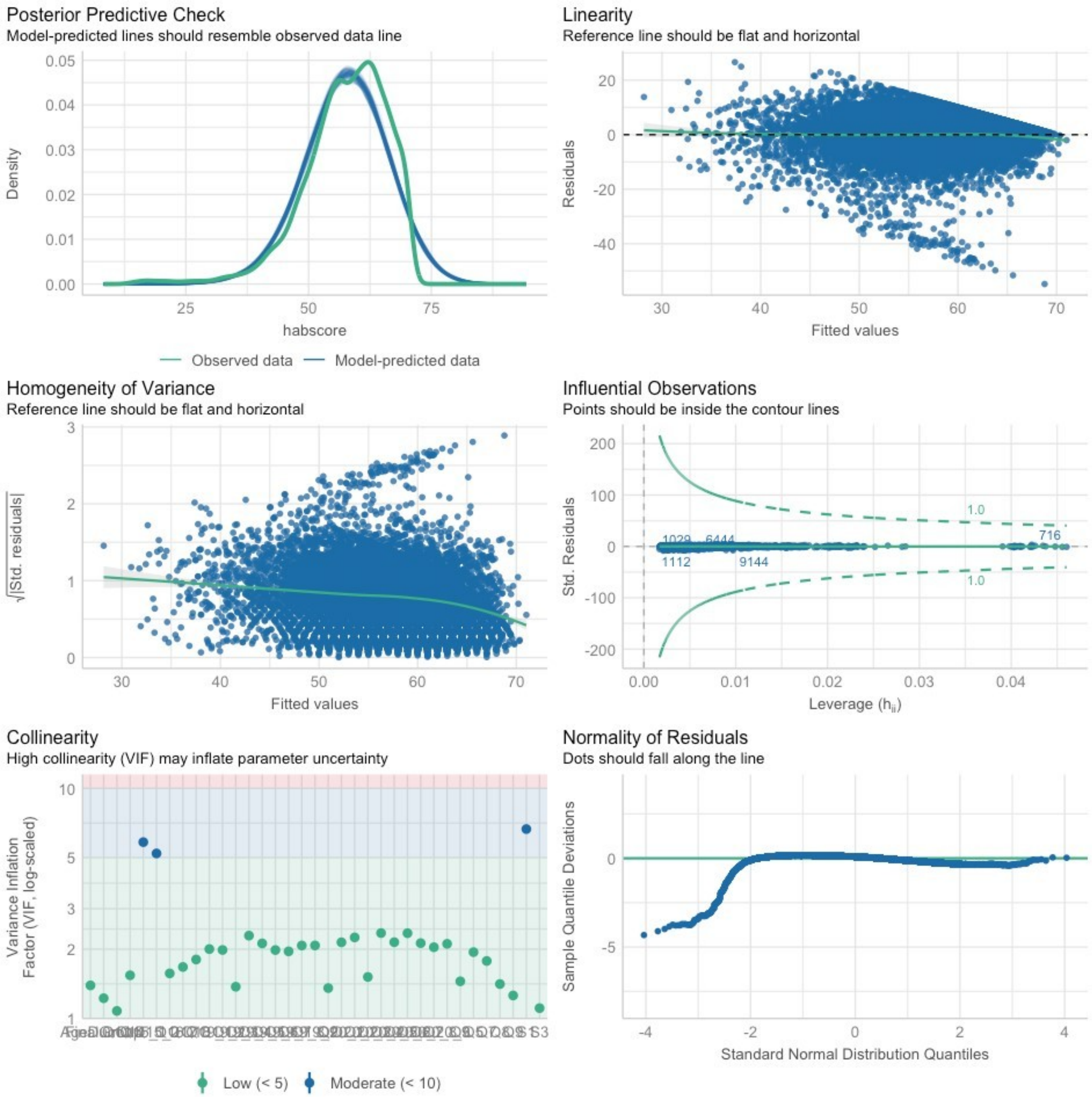

3.5 Models comparison

Hide

```
compare_performance(m1, m2, m3, rank = TRUE, verbose = FALSE)
```

```
# Comparison of Model Performance Indices

Name | Model |    R2 | R2 (adj.) |  RMSE | Sigma | AIC weights | AICc weights | BIC weights
| Performance-Score
-----------------------------------------------------------------------------------------------
-------------------
m3   |    lm | 0.192 |     0.190 | 7.754 | 7.765 |       0.977 |        0.974 |    1.73e-43
|           85.71%
m1   |    lm | 0.189 |     0.188 | 7.766 | 7.772 |       0.023 |        0.026 |        1.00
|           15.00%
m2   |    lm | 0.190 |     0.188 | 7.764 | 7.772 |    4.48e-05 |     4.78e-05 |    1.24e-25
|            6.90%
```

Hide

```
plot(compare_performance(m1, m2, m3, rank = TRUE, verbose = FALSE))
```

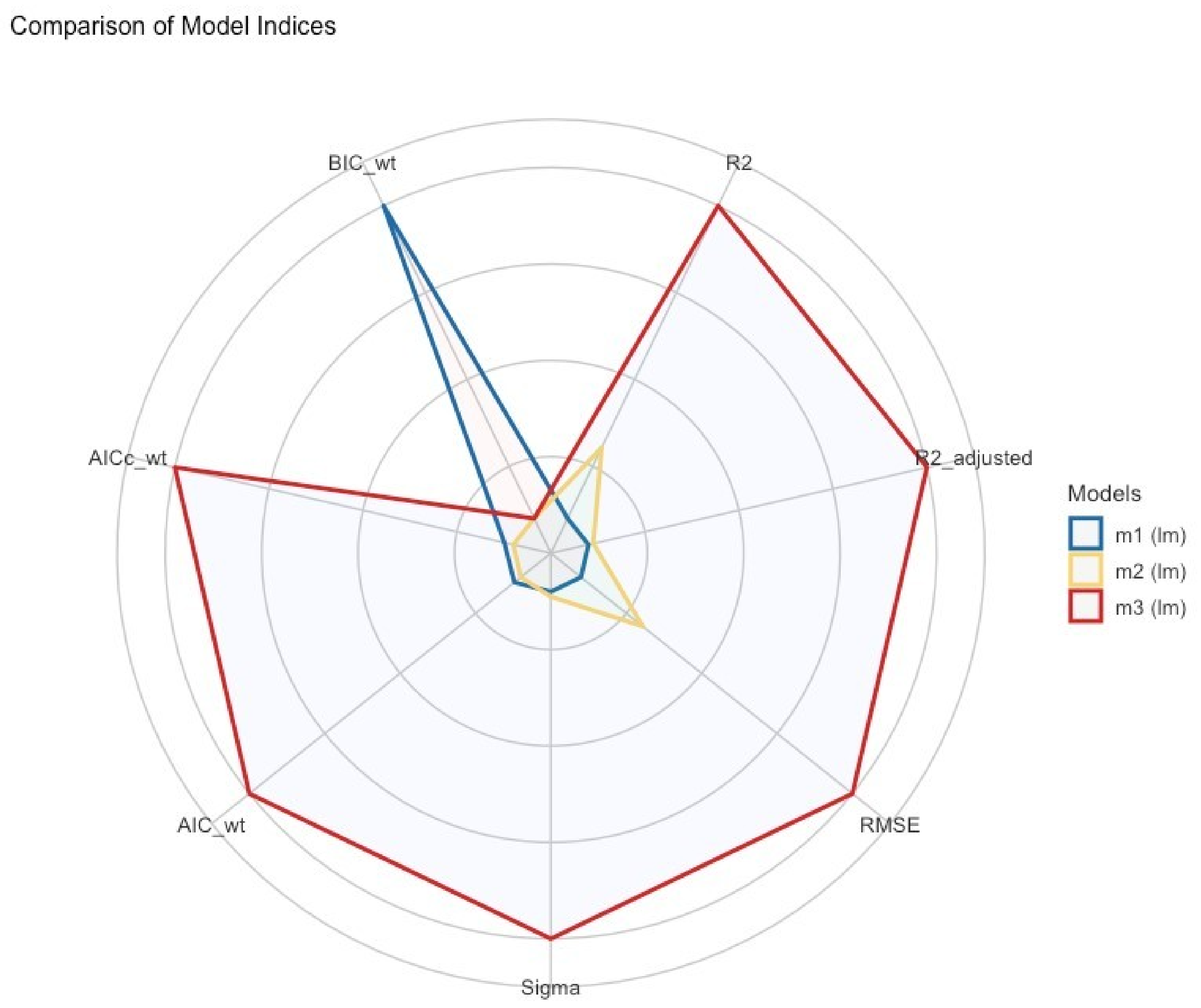

3.6 Testing models

Hide

```
test_performance(m1, m2, m3)
```

```
Name | Model |      BF
----------------------
m1   |    lm |
m2   |    lm | < 0.001
m3   |    lm | < 0.001
Each model is compared to m1.
```

Hide

```
test_bf(m1, m2, m3)
```

```
Bayes Factors for Model Comparison

     Model                                                   BF
[m2] S3 + AgeDum + FinalGroup + S1 + Q2 + Q3 + S3 * Q3 1.24e-25
[m3] S3 + AgeDum + FinalGroup + S1 + Q2 + Q3 + S3 * S1 1.73e-43

* Against Denominator: [m1] S3 + AgeDum + FinalGroup + S1 + Q2 + Q3
*   Bayes Factor Type: BIC approximation
```

1. c.dadousis@surrey.ac.uk (mailto:c.dadousis@surrey.ac.uk)↩
