# Supplementary material for: Companion animal owner “types” identified using a large-scale international assessment of the human-animal bond
Source: Front Vet Sci. 2026 May 12;13:1748135. doi: 10.3389/fvets.2026.1748135 (PMC13201171; doi:10.3389/fvets.2026.1748135)
Supplement: Supplementary file 3 [file Supplementary_file_3.docx]

**Supplementary File 3**

**
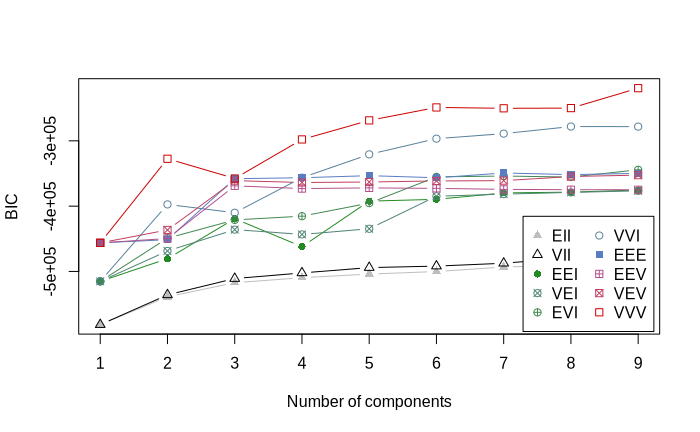
**

*Figure 1. Comparison of clusters within dog owners using Bayesian Information Criterion*

*
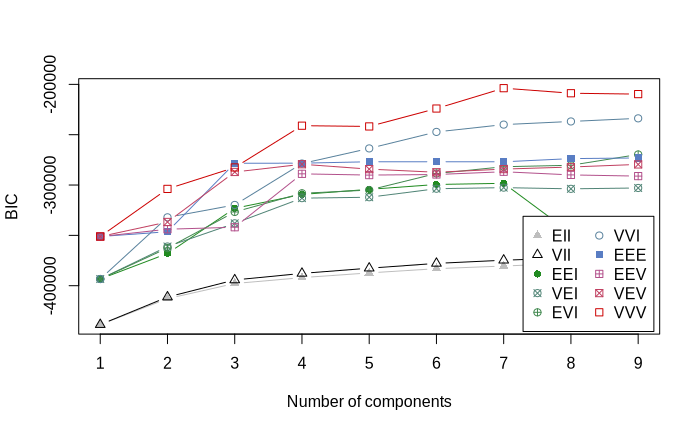
*

*Figure 2. Comparison of clusters within cat owners using Bayesian Information Criterion*
