# Supplementary material for: Companion animal owner “types” identified using a large-scale international assessment of the human-animal bond
Source: Front Vet Sci. 2026 May 12;13:1748135. doi: 10.3389/fvets.2026.1748135 (PMC13201171; doi:10.3389/fvets.2026.1748135)
Supplement: Supplementary file 4 [file Supplementary_file_4.docx]

**^Supplementary File 4^**

^Table 1: Results of Mann-Whitney-U tests comparing dog clusters^

| **Variable** | ***p*-value** | **Cluster 1 mean** | **Cluster 2 mean** |
| --- | --- | --- | --- |
| Age | <1e-4 | 2.94 | 3.08 |
| Gender | <1e-4 | 1.50 | 1.44 |
| Relationship of pet to owner | <1e-4 | 3.81 | 4.16 |
| Pet purpose | 1e-4 | 1.43 | 1.35 |
| Budget spent towards pet | <1e-4 | 2.15 | 2.35 |
| Use of flea medication | <1e-4 | 1.66 | 1.75 |
| Using nutritional supplements | <1e-4 | 1.25 | 1.28 |
| Using toothbrush | <1e-4 | 1.34 | 1.40 |
| Using dentist services | <1e-4 | 1.15 | 1.18 |
| Using annual check ups | <1e-4 | 1.57 | 1.67 |
| Using grooming services | <1e-4 | 1.48 | 1.59 |
| Using screening services | <1e-4 | 1.15 | 1.20 |
| Using none of the services | <1e-4 | 1.04 | 1.02 |
| Having health insurance | 0.80 | 1.79 | 1.79 |
| Using monitoring devices | 0.002 | 1.09 | 1.07 |
| Using osteoarthritis medication | 0.07 | 1.06 | 1.05 |
| Using medicine for skin-related disorders | 0.82 | 1.12 | 1.12 |
| Vet visits per year | <1e-4 | 4.30 | 4.45 |
| Pet has positive impact on owner’s health | <1e-4 | 2.80 | 2.87 |
| If pet impacts physical health, mental health, or both* | <1e-4 | 1.85 | 1.71 |
| Pet helps improve activity levels | <1e-4 | 1.43 | 1.53 |
| Pet helps provide comfort when owner is sad | <1e-4 | 1.44 | 1.61 |
| Pet helps calm owner when stressed | <1e-4 | 1.40 | 1.53 |
| Pet helps make owner happy | <1e-4 | 1.61 | 1.76 |
| Pet helps provide a greater sense of community | <1e-4 | 1.20 | 1.25 |
| Pet helps decrease owner’s loneliness | <1e-4 | 1.43 | 1.52 |
| Pet helps provide a sense of purpose | <1e-4 | 1.36 | 1.50 |
| Pet adds to owner’s happiness | <1e-4 | 1.56 | 1.72 |
| Pet has improved owner’s self-rating of health | <1e-4 | 1.35 | 1.50 |
| Medical doctor’s opinion that pet has improved health | 0.09 | 1.08 | 1.09 |
| Pet provides no help | <1e-4 | 1.02 | 1.01 |
| Importance of veterinarian knowledge | <1e-4 | 4.17 | 4.52 |
| Importance of veterinarian bond with pet | <1e-4 | 3.82 | 4.27 |
| Importance of veterinarian price | <1e-4 | 3.81 | 3.99 |
| Importance of veterinarian empathy towards pet | <1e-4 | 4.01 | 4.43 |
| Importance of veterinarian explanations | <1e-4 | 4.09 | 4.42 |
| Importance of veterinarian empathy towards owner | <1e-4 | 3.82 | 4.22 |
| Importance of veterinarian incorporating owner decisions | <1e-4 | 3.90 | 4.29 |
| Importance of veterinarian staff knowledge | <1e-4 | 3.99 | 4.38 |
| Importance of veterinarian environment | <1e-4 | 3.87 | 4,27 |
| Rating of veterinarian knowledge | <1e-4 | 3.36 | 3.57 |
| Rating of veterinarian bond with pet | <1e-4 | 3.19 | 3.45 |
| Rating of veterinarian price | <1e-4 | 2.93 | 3.08 |
| Rating of veterinarian empathy towards pet | <1e-4 | 3.27 | 3.50 |
| Rating of veterinarian explanations | <1e-4 | 3.33 | 3.53 |
| Rating of veterinarian empathy towards owner | <1e-4 | 3.21 | 3.44 |
| Rating of veterinarian incorporating owner decisions | <1e-4 | 3.25 | 3.49 |
| Rating of veterinarian staff | <1e-4 | 3.24 | 3.46 |
| Rating of veterinarian environment | <1e-4 | 3.20 | 3.42 |

^Key: *For this variable, a selected value of 1 signifies both mental and physical health improved, a selected value of 2 signifies mental health only, and a selected value of 3 signifies physical health only^

^Table 2: Results of Krusukal-Wallis and pairwise comparisons between cat clusters^

| **Variable** | **Kruskal-Wallis *p*-value** | **Cluster 1 mean** | **Cluster 2 mean** | **Cluster 3 mean** |
| --- | --- | --- | --- | --- |
| Age | <1e-4 | 2.95 | 3.04^3^ | 3.17 |
| Gender | <1e-4 | 1.42^1,2^ | 1.00^3^ | 2.00 |
| Relationship of pet to owner | <1e-4 | 3.37^1,2^ | 4.11^3^ | 3.76 |
| Pet purpose | <1e-4 | 1.48 | 1.34^3^ | 1.39 |
| Budget spent towards pet | <1e-4 | 2.08^1,2^ | 2.26 | 2.25 |
| Use of flea medication | <1e-4 | 1.51^1^ | 1.61 | 1.57 |
| Using nutritional supplements | <1e-4 | 1.15^2^ | 1.20 | 1.23 |
| Using toothbrush | 0.01 | 1.13 | 1.15 | 1.17 |
| Using dentist services | 1.1e-4 | 1.06 | 1.09 | 1.11 |
| Using annual check ups | <1e-4 | 1.41^1,2^ | 1.53 | 1.52 |
| Using grooming services | 2.5e-4 | 1.27^1^ | 1.35 | 1.34 |
| Using screening services | 1.1e-4 | 1.09^2^ | 1.14 | 1.15 |
| Using none of the services | <1e-4 | 1.16^1,2^ | 1.10 | 1.10 |
| Having health insurance | <1e-4 | 1.54^2^ | 1.59^3^ | 1.74 |
| Using monitoring devices | 6.5e-4 | 1.08 | 1.07 | 1.09 |
| Using osteoarthritis medication | 0.09 | 1.03 | 1.03 | 1.04 |
| Using medicine for skin-related disorders | 0.10 | 1.06 | 1.07 | 1.08 |
| Vet visits per year | <1e-4 | 3.58^2^ | 3.65^3^ | 3.82 |
| Pet has positive impact on owner’s health | <1e-4 | 2.61^1,2^ | 2.83 | 2.79 |
| If pet impacts physical health, mental health, or both* | <1e-4 | 1.91 | 1.84^3^ | 1.91 |
| Pet helps improve activity levels | 0.02 | 1.17 | 1.20 | 1.21 |
| Pet helps provide comfort when owner is sad | <1e-4 | 1.36^1^ | 1.58^3^ | 1.43 |
| Pet helps calm owner when stressed | <1e-4 | 1.39^1^ | 1.55^3^ | 1.44 |
| Pet helps make owner happy | <1e-4 | 1.48^1,2^ | 1.72^3^ | 1.61 |
| Pet helps provide a greater sense of community | 0.003 | 1.11 | 1.10 | 1.13 |
| Pet helps decrease owner’s loneliness | <1e-4 | 1.37^1^ | 1.54^3^ | 1.43 |
| Pet helps provide a sense of purpose | <1e-4 | 1.28^1,2^ | 1.47^3^ | 1.40 |
| Pet adds to owner’s happiness | <1e-4 | 1.44^1,2^ | 1.66^3^ | 1.57 |
| Pet has improved owner’s self-rating of health | <1e-4 | 1.27^1,2^ | 1.41^3^ | 1.35 |
| Medical doctor’s opinion that pet has improved health | 7.9e-4 | 1.07 | 1.07 | 1.09 |
| Pet provides no help | <1e-4 | 1.09^1,2^ | 1.01 | 1.02 |
| Importance of veterinarian knowledge | <1e-4 | 4.09^1^ | 4.40^3^ | 4.20 |
| Importance of veterinarian bond with pet | <1e-4 | 3.69^1^ | 4.10^3^ | 3.86 |
| Importance of veterinarian price | <1e-4 | 3.79 | 3.95^3^ | 3.81 |
| Importance of veterinarian empathy towards pet | <1e-4 | 3.92^1^ | 4.30^3^ | 4.03 |
| Importance of veterinarian explanations | <1e-4 | 4.02^1^ | 4.30^3^ | 4.06 |
| Importance of veterinarian empathy towards owner | <1e-4 | 3.75^1^ | 4.08^3^ | 3.89 |
| Importance of veterinarian incorporating owner decisions | <1e-4 | 3.85^1^ | 4.15^3^ | 3.94 |
| Importance of veterinarian staff knowledge | <1e-4 | 3.95^1^ | 4.21^3^ | 4.06 |
| Importance of veterinarian environment | <1e-4 | 3.79^1^ | 4.11^3^ | 3.94 |
| Rating of veterinarian knowledge | <1e-4 | 3.29^1^ | 3.48^3^ | 3.38 |
| Rating of veterinarian bond with pet | <1e-4 | 3.09^1^ | 3.34^3^ | 3.20 |
| Rating of veterinarian price | <1e-4 | 2.88 | 3.00 | 2.93 |
| Rating of veterinarian empathy towards pet | <1e-4 | 3.19^1^ | 3.41^3^ | 3.27 |
| Rating of veterinarian explanations | <1e-4 | 3.23^1^ | 3.44^3^ | 3.31 |
| Rating of veterinarian empathy towards owner | <1e-4 | 3.10^1^ | 3.34^3^ | 3.21 |
| Rating of veterinarian incorporating owner decisions | <1e-4 | 3.16^1^ | 3.39^3^ | 3.25 |
| Rating of veterinarian staff | <1e-4 | 3.14^1^ | 3.35^3^ | 3.25 |
| Rating of veterinarian environment | <1e-4 | 3.11^1^ | 3.32^3^ | 3.23 |

Key: ^1^significant at 3.7e-4 level for pairwise comparison Cluster 1 and Cluster 2; ^2^significant at 3.7e-4 level for pairwise comparison Cluster 1 and Cluster 3; ^3^significant at 3.7e-4 level for pairwise comparison Cluster 2 and Cluster 3; ^*For this variable, a selected value of 1 signifies both mental and physical health improved, a selected value of 2 signifies mental health only, and a selected value of 3 signifies physical health only^
