# Supplementary material for: Companion animal owner “types” identified using a large-scale international assessment of the human-animal bond
Source: Front Vet Sci. 2026 May 12;13:1748135. doi: 10.3389/fvets.2026.1748135 (PMC13201171; doi:10.3389/fvets.2026.1748135)
Supplement: Supplementary file 6 [file Supplementary_file_6.docx]

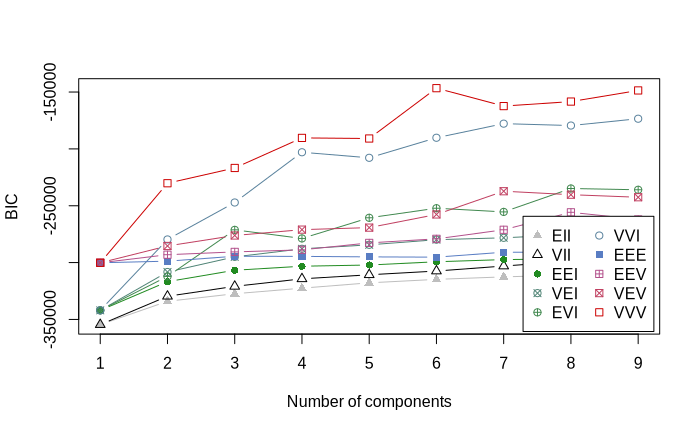


*Figure 1. Sensitivity analysis comparison of clusters within cat owners using Bayesian Information Criterion using only Human-Animal Bond Score questions and age*


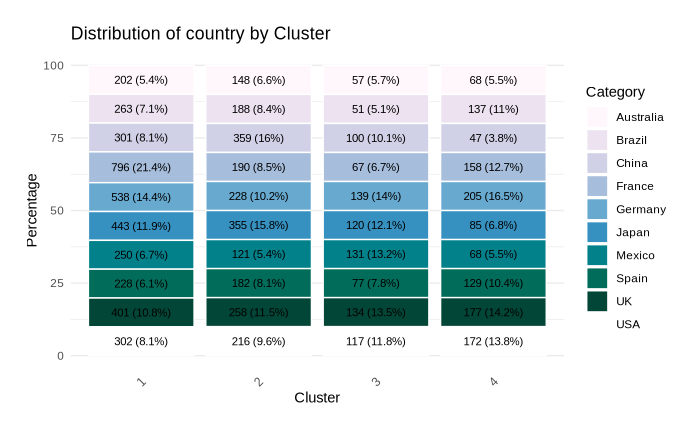


*Figure 2. Country distribution across clusters of cat owners identified using only Human-Animal Bond Score questions and age*


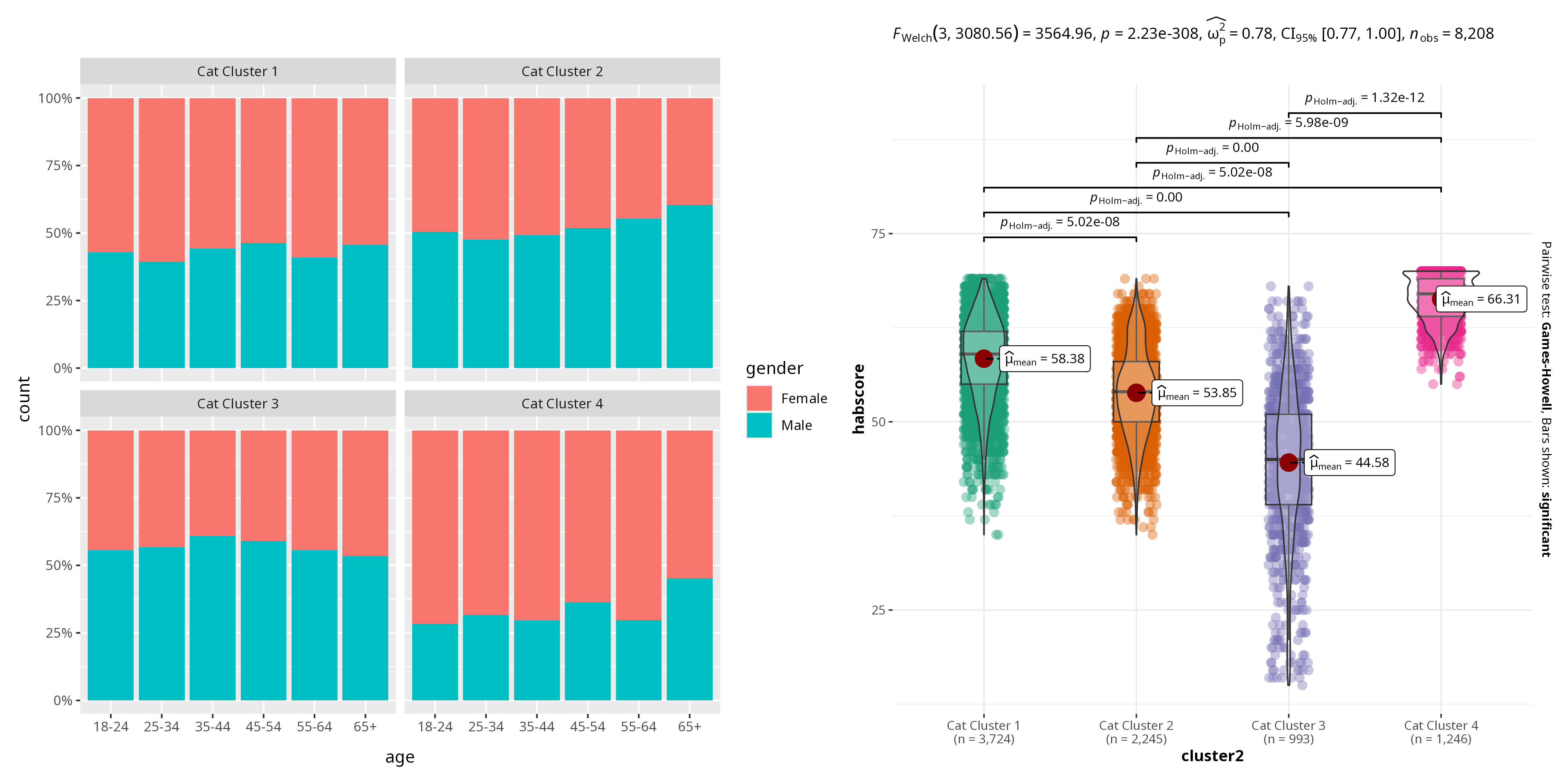


*Figure 3. Left – Proportion of gender of companion animal guardian with respect to age categories in the four cat clusters identified in this study; Right – Boxplots of the calculated HABSCORE in the four cat owner clusters. Results from sensitivity analysis using only Human-Animal Bond Score questions and age*
